# Rayleigh-Based Distributed Optical Fiber Sensing

**DOI:** 10.3390/s22186811

**Published:** 2022-09-08

**Authors:** Luca Palmieri, Luca Schenato, Marco Santagiustina, Andrea Galtarossa

**Affiliations:** 1 Department of Information Engineering, University of Padova, 35131 Padova, Italy; 2 CNIT, National Inter-University Consortium for Telecommunications, 43124 Parma, Italy; 3 Research Institute for Geo-Hydrological Protection, National Research Council, 35127 Padova, Italy

**Keywords:** Rayleigh scattering, fiber optics, sensor, distributed, polarization, pressure, structural health, double scattering

## Abstract

Distributed optical fiber sensing is a unique technology that offers unprecedented advantages and performance, especially in those experimental fields where requirements such as high spatial resolution, the large spatial extension of the monitored area, and the harshness of the environment limit the applicability of standard sensors. In this paper, we focus on one of the scattering mechanisms, which take place in fibers, upon which distributed sensing may rely, i.e., the Rayleigh scattering. One of the main advantages of Rayleigh scattering is its higher efficiency, which leads to higher SNR in the measurement; this enables measurements on long ranges, higher spatial resolution, and, most importantly, relatively high measurement rates. The first part of the paper describes a comprehensive theoretical model of Rayleigh scattering, accounting for both multimode propagation and double scattering. The second part reviews the main application of this class of sensors.

## 1. Introduction

The idea of using optical fibers as sensors is almost as old as the idea of using them for telecommunications [1], yet it took several decades before significant technological progress in both photonics and electronics made optical fiber sensors effective and viable tools, spurring intense research activities and paving the way for many applications.

Optical fiber sensors (OFSs) offer several advantages with respect to standard ones, including long operational range, ease of multiplexing, small form factor, immunity to electromagnetic interference, and robustness to extreme temperatures; among the several OFS flavors, distributed optical fiber sensors (DOFSs) are unique tools unparalleled by any other technology [2]. DOFSs exploit one of the three possible scattering mechanisms occurring in optical fibers, i.e., Raman, Brillouin, and Rayleigh. By probing the optical fiber with well-tailored light and measuring the resulting small amount of backscattered power over time, DOFSs can perform a distributed measurement of the fiber’s local properties. In this way, a fiber strand is converted into a concatenation of independent sensors that can be addressed individually, enabling the mapping of various physical parameters along the path where the fiber is deployed. The number of these sensing points can easily exceed a few tens of thousands, distributed over distances that can range from a few meters to many tens of kilometers.

Depending on whether it is based on Raman, Brillouin, or Rayleigh scattering, the DOFS has different characteristics. Specifically, Raman scattering is the only one being sensitive to only one physical parameter: temperature [2,3,4,5,6]. Depending on the point of view, this is either an advantage because it avoids cross-sensitivity to other physical fields or a disadvantage because it limits the applicability to temperature sensing. Differently, Brillouin scattering is intrinsically sensitive to both temperature and strain [2,4,7,8,9], making the technique more versatile, on the one hand, but at the same time prone to cross-sensitivity, on the other hand. Both Raman and Brillouin scattering are nonlinear inelastic processes; therefore, they require high probing energy and are plagued by relatively low efficiency. As a result, Raman and Brillouin measurements have typically low SNR, which leads to relatively long measurement times—in the order of several seconds if not many minutes. On the other hand, Rayleigh scattering offers somewhat higher SNR, to the extent that single-shot, high-repetition-rate measurements are possible, enabling a fundamental and rather unique feature such as distributed acoustic sensing (DAS) [2,10,11].

Another characteristic feature of Rayleigh scattering is that it is *per se* independent of almost any external physical field [12,13]. In fact, while Brillouin and Raman scattering are intrinsically dependent on strain and/or temperature, in Rayleigh-based DOFSs, the scattering itself is used only to track and reveal propagation effects, which are the actual sensing mechanisms. More specifically, Rayleigh scattering can be thought of as being generated by isolated scattering centers, randomly distributed along the fiber. Each center does not have an intrinsic sensitivity to the external environment, yet they act as *mirrors* back-reflecting the probing light to the fiber input; the sensing ability is then achieved by analyzing the effects that the environment has induced on the propagation of light. The most effective mechanism relies on the interference between light reflected by different scattering centers: as the relative position of these centers varies, so do the interference fringes, enabling the sensing of temperature and/or strain variations [14]. Similarly, perturbations acting on the fiber can influence the polarization of the propagating light so that by analyzing the state of polarization of the backscattered light, it is possible to perform distributed sensing of parameters such as twist, magnetic fields, and electric current [15].

Rayleigh-based DOFSs come in two main flavors: optical time-domain reflectometry (OTDR) and optical frequency-domain reflectometry (OFDR). In OTDR, a single light pulse is sent into the fiber, while the generated backscattered light is measured over time. The shorter the pulse, the higher the spatial resolution, yet the lower the SNR. At the same time, the pulse peak power cannot be increased above a certain threshold to avoid the onset of disruptive nonlinear phenomena [16]. These constraints limit the spatial resolution of OTDRs in the range of about a meter over several tens of kilometers. While OTDR measures the round-trip impulse response of the fiber, OFDR measures its round-trip frequency response, i.e., the Fourier transform of the impulse response [17]. Actually, the OFDR probes the fiber with frequency-swept CW light and measures the beating tone between the backscattered light and a portion of the probe light. The spatial resolution of distributed sensors is inversely proportional to the bandwidth of the probe light; because of this, the OFDR can achieve sub-millimeter spatial resolution by scanning bandwidths of tens of nanometers. Nevertheless, the sensing range is limited by the coherence of the light source. It typically does not exceed a few hundred meters—although complex phase-compensation schemes can push this limit in the several kilometers range [18,19,20].

This paper reviews the theory and applications of Rayleigh-based distributed sensing. Section 2 presents a comprehensive and original theoretical model of Rayleigh scattering. The model is based on the formalism of scattering matrices and includes multimode propagation and double scattering. Indeed, there is a recent increasing interest in OFS based on multimode or multi-core fibers [21,22,23]; moreover, double scattering may play a nonnegligible role in scattering-enhanced fibers [22,24]. This general theory is specialized to distributed sensing in single-mode fibers in Section 3, where the main sensing mechanisms are reviewed. Many of the proofs and details and these theoretical sections are deferred to the extensive Appendices. Finally, Section 4 reviews the most recent applications of Rayleigh-based DOFSs.

## 2. Phenomenological Model of Distributed Measurements in Optical Fibers

In this section, we derive a phenomenological model of distributed measurements based on Rayleigh scattering in optical fibers; we begin considering generic multimode fibers and then specialize the theory to single-mode ones. The only starting assumption is that the optical fiber is a linear device. It should also be noted that the theory also applies to multi-core fibers, as long as the propagation is described in terms of super-modes.

**Figure 1:**
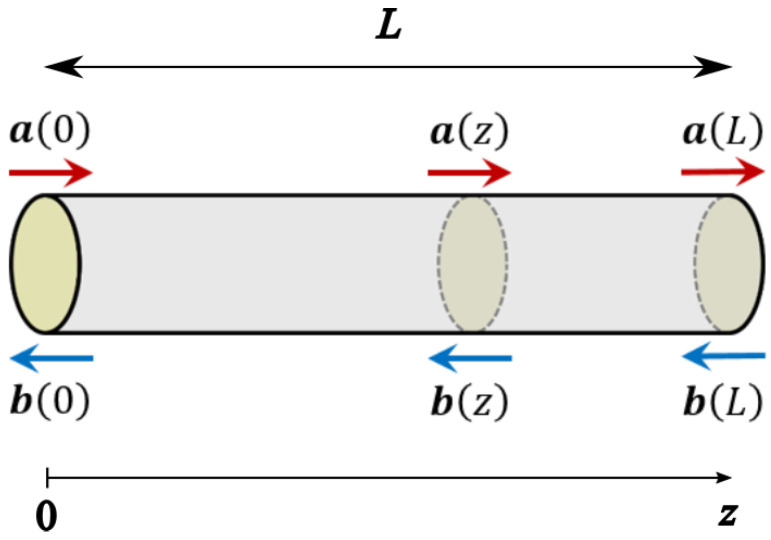
Schematics of the field definitions.

An optical fiber supporting the propagation of *N* modes (counting both polarization and spatial ones) can be considered as a 2N-port device and described by its scattering matrix. We introduce the longitudinal coordinate *z* with z=0 at one facet of the fiber and z=L at the other facet; then we indicate with a(z) the *N*-dimensional vector representing the complex amplitudes of the *forward* propagating modes and with b(z) the analogous vector for the *backward* propagating ones (see Figure 1). Accordingly, the 2N-dimensional vectors representing the modes that *enter* and *exit* the fiber facets are, respectively,

(1) $$E_{\mathrm{in}}=\left[\begin{array}{c} a(0)\\ b(L) \end{array}\right]\;,\;E_{\mathrm{out}}=\left[\begin{array}{c} b(0)\\ a(L) \end{array}\right]\;;$$

correspondingly, the 2N×2N scattering matrix S can be divided into N×N blocks such that [25]:

(2) $$E_{\mathrm{out}}=\left[\begin{array}{c} b(0)\\ a(L) \end{array}\right]=\left[\begin{array}{cc} R_{1}\left(L\right) & B(L)\\ F(L) & R_{2}\left(L\right) \end{array}\right]\left[\begin{array}{c} a(0)\\ b(L) \end{array}\right]=S\;E_{\mathrm{in}}\;.$$

Considering the case in which light is launched only at z=0—i.e., setting b(L)=0, we find a(L)=F(L)a(0), which confirms that F(L) is the matrix, actually the *Jones* matrix [26], describing *forward transmission* across the fiber. Under the same conditions we also find that $b\left(0\right)=R_{1}\left(L\right)a\left(0\right)$, so that $R_{1}\left(L\right)$ describes the reflection, or *round trip*, when launching from z=0. Similarly, in the case light is launched only from z=L, hence a(0)=0, we find b(0)=B(L)b(L), which clarifies that B(L) is the Jones matrix describing *backward propagation* across the fiber; finally, $R_{2}\left(L\right)$ is the “backward” round trip from the facet at z=L.

Before proceeding, it is worthwhile clarifying that the vectors $E_{\mathrm{in}}$ and $E_{\mathrm{out}}$, and hence a and b, represent the complex amplitudes of the modes at a specific frequency; basically, they are the phasors associated with each field component at that frequency. In other words, (2) must be interpreted in the frequency domain and all the quantities are functions of the angular frequency $\Omega =\omega -\omega_{0}$, where ω is the physical angular frequency and $\omega_{0}$ is the reference angular frequency (typically that of the source). From this perspective, F(L) is the spectral response of the fiber with respect to forward propagation and, similarly, $R_{1}\left(L\right)$ is its round trip spectral response. The dependence on Ω is not explicitly reported for the sake of brevity.

The structure of the scattering matrices also reflects other possible physical properties of the device. In particular, S is symmetric if and only if the device is *reciprocal* [25]; accordingly, we can conclude that for a reciprocal fiber

(3) $$B\left(L\right)=F^{T}\left(L\right)\;,\;R_{1}\left(L\right)=R_{1}^{T}\left(L\right)\;,\;R_{2}\left(L\right)=R_{2}^{T}\left(L\right)\;.$$

This confirms, and extends to the *N*-dimensional case, the well known property that the backward Jones matrix of a reciprocal fiber is equal to the transpose of the forward Jones matrix [27]. Moreover, it proves that the round trip matrices must be symmetric for any reciprocal device.

### 2.1. Propagation along the Fiber

Equation (2) describes the transmission across the whole fiber; however, having in mind distributed measurements, we are interested in describing the propagation *along* the fiber. To this aim, we can write the following scattering relation

(4) $$\left[\begin{array}{c} b(0)\\ a(z) \end{array}\right]=\left[\begin{array}{cc} R_{1}\left(z\right) & B(z)\\ F(z) & R_{2}\left(z\right) \end{array}\right]\left[\begin{array}{c} a(0)\\ b(z) \end{array}\right]\;,$$

which relates the fields entering in and exiting from a fiber section comprised between z=0 and the generic position *z* (see Figure 1). Similarly to before, F(z) represents forward propagation up to *z* and analogous interpretations can be given to the other matrices. Note, however, that b(z) is the field backward propagating *inside* the fiber at *z*; therefore, we cannot set it arbitrarily to 0 as we can do for b(L), because it depends on what happens in the fiber section beyond the point *z*. Actually, according to (4), the forward propagating field at *z* reads $a\left(z\right)=F\left(z\right)a\left(0\right)+R_{2}\left(z\right)b\left(z\right)$, where the first term is the contribution of forward propagation, whereas the second term describes the reflection of the backward propagating field. Assuming that no light was launched at z=L, i.e., assuming that b(L)=0, we can conclude that $R_{2}\left(z\right)$ represents the *double reflection* of the field a(0) launched at z=0; more specifically to optical fibers, $R_{2}\left(z\right)$ represents *double Rayleigh scattering*.

To describe the dependence on *z* of the vectors a(z) and b(z), we can rearrange (4) so that the fields at *z* are expressed as functions of the fields at z=0; after some algebra, we can write

(5) $$\left[\begin{array}{c} a(z)\\ b(z) \end{array}\right]=\left[\begin{array}{cc} F-R_{2}B^{-\;1}R_{1} & R_{2}B^{-\;1}\\ -B^{-\;1}R_{1} & B^{-\;1} \end{array}\right]\left[\begin{array}{c} a(0)\\ b(0) \end{array}\right]\;,$$

where the explicit dependence on *z* is omitted for compactness of the notation. Taking the *z*-derivative of (5) we find (see Appendix C)

(6) $$\left[\begin{array}{c} \partial_{z}a\\ \partial_{z}b \end{array}\right]=\left[\begin{array}{cc} \left(\partial_{z}F\right)F^{-\;1}-R_{2}B^{-\;1}\left(\partial_{z}R_{1}\right)F^{-\;1} & W\\ -B^{-\;1}\left(\partial_{z}R_{1}\right)F^{-\;1} & -B^{-\;1}\partial_{z}B+B^{-\;1}\left(\partial_{z}R_{1}\right)F^{-\;1}R_{2} \end{array}\right]\left[\begin{array}{c} a\\ b \end{array}\right]\;,$$

with

(7) $$W=\partial_{z}R_{2}-\left(\partial_{z}F\right)F^{-\;1}R_{2}-R_{2}B^{-\;1}\partial_{z}B+R_{2}B^{-\;1}\left(\partial_{z}R_{1}\right)F^{-\;1}R_{2}\;,$$

where $\partial_{z}$ represents partial derivative with respect to *z*. Cumbersome as it is, Equation (6) describes the forward and the backward propagating fields in the most general case of a nonreciprocal fiber, including the effects of double scattering. The equation gets substantially simpler when double scattering can be neglected—i.e., when $R_{2}\approx 0$ —in which case the element W given by (7) becomes zero.

It is worthwhile noticing that Equation (6) is mathematically equivalent to the equation provided by coupled-mode theory (CMT) [28,29]. Actually, in the next section we show how the specific expressions of F, B, $R_{1}$ and $R_{2}$ depend on the physical properties of the fiber and can be calculated by CMT.

### 2.2. Coupled-Mode Representation

Coupled mode theory (CMT) allows us to write a differential equation for the complex amplitudes of the forward and the backward propagating modes [28,29]. In general, this equation can be written as (again we omit the dependence on *z* for brevity)

(8) $$\left[\begin{array}{c} \partial_{z}a\\ \partial_{z}b \end{array}\right]=-j\left(\left[\begin{array}{cc} \beta & 0\\ 0 & -\beta \end{array}\right]+\left[\begin{array}{cc} K & S_{2}\\ S_{1} & C \end{array}\right]\right)\left[\begin{array}{c} a\\ b \end{array}\right]=-j\left[\begin{array}{cc} Q & S_{2}\\ S_{1} & H \end{array}\right]\left[\begin{array}{c} a\\ b \end{array}\right]\;,$$

where Q(z)=β+K(z), H(z)=−β+C(z), β is a diagonal matrix with the propagation constants of the forward propagating modes (possibly complex to account for losses), K(z) is the matrix accounting for coupling among the forward propagating modes and C(z) accounts for coupling among backward propagating ones. Matrices $S_{1}$ and $S_{2}$ are instead related to coupling between forward and backward propagating modes, i.e., to generic reflection processes, such as scattering. While having a more general meaning—consider for example the case of fiber Bragg gratings—hereinafter we refer to these matrices as describing Rayleigh scattering (we defer the reader to Appendix A.4 for a deeper discussion about the interpretation of those matrices).The elements of the above-mentioned matrices are related to the specific perturbations acting on the fiber [28,29]. Nevertheless, by comparing (8) with (6) we can draw some general conclusions.

Comparing the elements of position (2,1) we have $B^{-\;1}\left(\partial_{z}R_{1}\right)F^{-\;1}=jS_{1}$ and hence

(9) $$R_{1}\left(L\right)=j\int_{0}^{L}B\left(z\right)S_{1}\left(z\right)F\left(z\right)dz\;.$$

This is the main result of this paper, as it provides a general expression for the matrix describing round trip propagation (from z=0) across a generic nonreciprocal fiber, including also the effects of double scattering. Recalling that, as commented above, the equations must be interpreted in the frequency domain, we can conclude that $R_{1}\left(L\right)$ is the round trip frequency response of the fiber. Note that it depends explicitly only on the Rayleigh scattering matrix $S_{1}$, while double scattering is implicitly included in B and F. Actually, (9) cannot be evaluated as long as we do not know the forward and backward propagation matrices F(z) and B(z). To this aim, comparing elements of position (1,1) and noticing that $R_{2}B^{-\;1}\left(\partial_{z}R_{1}\right)F^{-\;1}=jR_{2}S_{1}$ we find

(10) $$\partial_{z}F=-j\left\{Q\left(z\right)-R_{2}\left(z\right)S_{1}\left(z\right)\right\}F\left(z\right)\;,\;F\left(0\right)=I.$$

This equation is a neat generalization including double scattering (which now appears explicitly) of the more widely known forward-propagation equation $\partial_{z}F=-jQ\;F$. Similar considerations on the elements (2,2) yield (mind the order)

(11) $$\partial_{z}B=jB\left(z\right)\left\{H\left(z\right)+S_{1}\left(z\right)R_{2}\left(z\right)\right\}\;,\;B\left(0\right)=I.$$

Finally, comparing the elements in position (1,2) we find the equation for $R_{2}$:

(12) $$\partial_{z}R_{2}=-j\left(Q\;R_{2}-R_{2}H-R_{2}S_{1}R_{2}+S_{2}\right)\;,\;R_{2}\left(0\right)=0\;,$$

which is a matrix Riccati differential equation [30].

In order to study Rayleigh-based distributed measurements we should calculate the matrix $R_{1}\left(L\right)$ given by (9). According to the above analysis, to achieve this result we first have to solve Equation (12) and then Equations (10) and (11); details about how these numerical calculations can be carried out are reported in Appendix B. As mentioned above, double scattering may play a nonnegligible role in scattering-enhanced fibers, and the model just introduced enables tackling this problem. Nonetheless, double scattering can be neglected in the most common scenarios, where we can safely assume that $R_{2}\left(z\right)\approx 0$ and drastically simplify the mathematical framework.

### 2.3. The Role of External Perturbations

The expressions of the matrices K, C, $S_{1}$ and $S_{2}$ depend on the fiber characteristics and on the external perturbations acting on the fiber itself. To begin with, there are specific properties related to reciprocity and losslessness. Namely, as shown in Appendix C, if the fiber is reciprocal it must be

(13) $$C\left(z\right)=-K^{T}\left(z\right)\;,\;S_{1}\left(z\right)=S_{1}^{T}\left(z\right)\;,\;S_{2}\left(z\right)=S_{2}^{T}\left(z\right)\;,$$

which also implies $H\left(z\right)=-Q^{T}\left(z\right)$. Differently, if the fiber is lossless it must be

(14) $$Q\left(z\right)=Q^{*}\left(z\right)\;,\;H\left(z\right)=H^{*}\left(z\right)\;,\;S_{2}\left(z\right)=-S_{1}^{*}\left(z\right)\;,$$

which implies that β is real and K(z) and C(z) are Hermitian.

More in general, the elements of K(z) and C(z) can be calculated by proper overlap integrals of the modes’ fields over the fiber cross-section, involving the external perturbations such as bending, twist, etc. (see Refs. [28,29] and Appendix A). When more perturbations act at the same time, K can be expressed as

(15) $$K\left(z\right)=\sum_{n}K_{n}\left(z\right)\;,$$

where $K_{n}$ is the coupling matrix of each perturbation when acting alone [31,32]. It is important to remark that, as long as the fiber is not twisted and it is not exposed to an external magnetic field inducing Faraday rotation, the matrix K must be real [33,34] (see Appendix A).In other words, only by twisting the fiber or by inducing Faraday rotation, K can be made complex. This is a generalization to multimode fibers of the fact that only twist and Faraday rotation induce circular birefringence [32,35,36], and it has important practical consequences for sensing applications, as discussed in the following sections. Similar considerations can be made about C(z), the coupling matrix of the backward propagating modes.

As already noted, for reciprocal fibers it must be $C=-K^{T}$. Regarding nonreciprocal fibers, note that only Faraday rotation can break reciprocity [36]. As recalled in Appendix A.2, if $K_{\mathrm{faraday}}$ is the coupling matrix for forward propagating modes due to Faraday rotation, then the corresponding matrix for the backward propagating ones is $C_{\mathrm{faraday}}=-K_{\mathrm{faraday}}$. In conclusion, the general coupling matrices for forward and backward propagating modes can be written, respectively, as

(16) $$\begin{array}{cc} K(z) & =K_{\mathrm{linear}}+jK_{\mathrm{twist}}+jK_{\mathrm{faraday}}\;,\\ C(z) & =-K_{\mathrm{linear}}^{T}+jK_{\mathrm{twist}}-jK_{\mathrm{faraday}}\;, \end{array}$$

where all the terms on the right-hand side are *real*, $K_{\mathrm{twist}}$ is the anti-symmetric coupling matrix due to the twist (see Appendix A.1) and $K_{\mathrm{linear}}$ is the coupling due to all the other perturbations, such as bending, lateral pressure, etc., that cause effects similar to linear birefringence [33].

### 2.4. Invariance of the Round Trip Response with Respect to a Local Rotation of the Reference Frame

The round trip spectral response $R_{1}\left(L\right)$ has a property with a remarkable impact on distributed measurements and sensing. Specifically, $R_{1}\left(L\right)$ is invariant with respect to a rotation of the reference frame by an angle θ(z) around the fiber axis, provided that θ(0)=0—i.e., the rotation is null at the beginning of the fiber.

The mathematical proof of this property is given in Appendix C.4; here we provide a simpler, yet more insightful, physical motivation. The point is that the quantity $b\left(0\right)=R_{1}\left(L\right)a\left(0\right)$ is the spectral round trip response of the fiber, measured with respect to the reference frame set at the fiber input facet, that is at z=0. Any rotation of the reference frame *along* the fiber is just a mental construction of the experimenter, which has not any physical consequences on the measurement, as long as the *measurement* reference frame is not changed—i.e. as long as θ(0)=0.

While having no physical consequences, this invariance does have consequences on the interpretation of distributed measurements. Actually, it can be proved (see Appendix C.4 for details) that the rotation of the reference frame along the fiber can be chosen in such a way as to convert the coupling due to the torsional stress induced by the twist in an apparent rotation of the linear coupling. More specifically, in the new rotated frame, the coupling matrices for forward and backward propagating modes read, respectively,

(17) $$\begin{array}{cc} \tilde{K}\left(z\right) & =TK_{\mathrm{linear}}T^{T}+jK_{\mathrm{faraday}}\;,\\ \tilde{C}\left(z\right) & =-TK_{\mathrm{linear}}^{T}T^{T}-jK_{\mathrm{faraday}}\;, \end{array}$$

where the transformation matrix T(z) is given by

(18) $$\partial_{z}T=-K_{\mathrm{twist}}\left(z\right)T\left(z\right)\;,\;T\left(0\right)=I\;.$$

The invariance of $R_{1}\left(L\right)$ with respect to the rotation of the reference frame means that the round trip measurement performed on a fiber described by the coupling matrices (16) can be interpreted as if the measurement were taken on a fiber with apparent coupling matrices as in (17). As a consequence, the imaginary part of the measured $\tilde{K}\left(z\right)$ can be attributed to Faraday rotation only, enabling the distributed measurement of magnetic fields, whereas any variation of the fiber twist results in a known transformation of the linear coupling $K_{\mathrm{linear}}$, enabling the distributed measurement of fiber rotation. Examples of these noticeable sensing applications are given in Section 4.5 and Section 4.6.

## 3. Distributed Sensing in Single-Mode Fibers

We now specialize the above theory to the case of single-mode fibers, where the only propagating mode is the fundamental one LP_0,1_. Rayleigh scattering in optical fibers has been studied in several papers [37,38]; here, following the phenomenological approach, we describe it using the CMT as formulated by Marcuse [28]. Accordingly, the origin of Rayleigh scattering can be described by a scalar real random fluctuation, Δε, of the dielectric constant. As shown in Appendix A.3, the scattering matrices can then be expressed as

(19) $$S_{1}\left(z\right)=\eta \left(z\right)I=-S_{2}\left(z\right)\;,$$

where η(z) is the backscattering coefficient *per unit length*, which is proportional to Δε (see Equation (A16)). The fact that the matrices are proportional to the identity matrix I confirms the common knowledge that Rayleigh backscattering is co-polarized with the exciting field, when both are represented with respect to the same reference frame, as always performed in this paper; in other words, the *x*-polarized LP_0,1_ mode does not induce scattering on the *y*-polarized modes, and vice versa. For completeness, we note that this result is to some extent an approximation since the random fluctuations of the dielectric permittivity in fused silica can be slightly anisotropic; therefore, a small coupling between orthogonal polarization can be expected [2]. The effect is, however, rather small and commonly neglected. The random fluctuations Δε occur on a spatial scale much smaller than the wavelength, hence with a very short spatial correlation. Accordingly, η(z) can be reasonably modeled as a zero-average, delta-correlated random spatial process.

The forward spectral response F can be factorized as (we recall that $\Omega =\omega -\omega_{0}$)

(20) $$F\left(z,\Omega \right)=e^{-\alpha (z)}e^{-j\beta (\Omega )z}U\left(z,\Omega \right)\;,$$

where α accounts for the attenuation, β is the propagation constant, and U is a matrix accounting only for polarization effects. In single-mode fibers, polarization dependent loss (PDL) is largely negligible [39], therefore U is unitary with unit determinant. Similarly,

(21) $$B\left(z,\Omega \right)=e^{-\alpha (z)}e^{-j\beta (\Omega )z}V\left(z,\Omega \right)\;,$$

where V is unitary because of no PDL; in the case of reciprocal fibers $V=U^{T}$. In single-mode fibers the round trip spectral response (9) can then be rearranged as

(22) $$R_{1}\left(L,\Omega \right)=j\int_{0}^{L}\eta \left(z\right)e^{-2\alpha (z)}e^{-j2\beta (\Omega )z}V\left(z,\Omega \right)U\left(z,\Omega \right)dz\;.$$

From the standpoint of distributed measurements what is most interesting is the round trip impulse response of the fiber, i.e., the Fourier transform of (22); to perform this step, we need first specify the frequency dependence of the involved quantities. The scattering coefficient η is proportional to the square of frequency, as typical of Rayleigh scattering; this effect can however be safely neglected over the optical band typically considered in a single distributed measurement. For similar reasons, the frequency dependence of the accumulated attenuation α can also be neglected. Differently, β and the matrices U and V include also dispersion effects, such as chromatic dispersion and polarization mode dispersion (PMD), which can impact the measurement. Nonetheless, all the techniques envisaged so far assume that these dispersion effects are negligible. This is equivalent to assuming that the propagation constant can be approximated as $\beta \left(\Omega \right)\approx \beta_{0}+\Omega \beta_{1}$, where $\beta_{0}=\beta \left(\omega_{0}\right)$ and $\beta_{1}=d\beta /d\omega =1/v_{g}$ is the inverse group velocity evaluated at $\omega_{0}$. Moreover, U and V are assumed to be independent of frequency, which is reasonable as long as the product of differential group delay and measurement bandwidth is much smaller than 1. Owing to these assumptions and taking the inverse Fourier transform of (22), the round trip impulse response can be expressed as

(23) $$R\left(t\right)=R\left(z_{t}\right)=j\frac{v_{g}}{2}\eta \left(z_{t}\right)e^{-\alpha (z_{t})}e^{-j2\beta_{0}z_{t}}V\left(z_{t}\right)U\left(z_{t}\right)\;,$$

where $z_{t}=v_{g}\;t/2$ is the position along the fiber corresponding to time *t*.

When a pulse with shape a(t) and polarization ${\hat{a}}_{0}$ is launched into a fiber, the corresponding round trip response can then be written as

(24) $$b\left(z_{t}\right)=\rho \left(z_{t}\right)e^{-\alpha (z_{t})}e^{-j2\beta_{0}z_{t}}V\left(z_{t}\right)U\left(z_{t}\right){\hat{a}}_{0}\;,$$

where

(25) $$\rho \left(z_{t}\right)=j\int_{z_{t}-\Delta /2}^{z_{t}}\eta \left(z\right)e^{-j2\beta_{0}\left(z-z_{t}\right)}a\left(t-2\beta_{1}z\right)dz\;,$$

Δ is the pulse length *in the fiber*, and we assumed that the variations of α(z), U(z) and V(z) are negligible over the length Δ/2. The quantity ρ is a complex random variable describing the amplitude and phase of the backscattered light. The integral over half the pulse length describes the interference among the scattering centers of the fiber illuminated by the pulse and hence, as discussed below, $\rho (z_{t})$ is the so-called *fingerprint* of the fiber.

Note that the above analysis has been performed having in mind an OTDR scheme. However, the validity is more general and includes also the OFDR scheme; in this case, it is enough to consider a(t) as the (very short) pulse resulting from the inverse Fourier transform of the (wide) spectrum of the probe light.

In principle, all three main physical parameters—namely attenuation, phase and polarization, can be used to perform distributed sensing. Nonetheless, despite some earlier distributed sensing systems being based on the measurement of local attenuation [40,41], nowadays the most successful ones do not consider this parameter; therefore, hereinafter we neglect it. On the contrary, the use of phase and polarization for distributed sensing is described in the following sections.

### 3.1. Phase-Based Distributed Sensing

Phase-based distributed sensing is intrinsically sensitive to variations of strain, Δϵ, and temperature, ΔT. This sensitivity occurs through the factors $e^{-j2\beta_{0}z}$ that appear in (24) and (25). Actually, the phase $\phi =\beta_{0}z$ can be expressed as $\phi =\omega_{0}n_{0}z/c_{0}$, where $n_{0}$ is the effective refractive index at $\omega_{0}$ and $c_{0}$ the speed of light in vacuum. It is well known that $n_{0}$ depends on temperature and strain variations, because of the thermo-optic and elasto-optic effects, respectively [42]. Similarly, also *z* depends on temperature and strain variations, because of the thermal and geometrical expansion/contraction, respectively [42]. Therefore, the phase ϕ varies with temperature and strain and it can be used to sense these parameters [43]. We should remark, however, that this cross-sensitivity raises practical issues, because it renders phase-based Rayleigh sensing unable to distinguish between temperature and strain variations, unless other information is available. Moreover, it should be noted that the phase ϕ also depends on the frequency $\omega_{0}$ of the laser source; therefore, any drift in this parameter yields a variation of the phase and a consequent misinterpretation of the sensor readout. These two problems lead to two main application scenarios.

In OTDR systems, where the laser is narrow-band, it is typically not easy to compensate for the frequency drift of the source. Nevertheless, since this drift occurs at relatively low frequencies (some Hertz or below), it does not impact the high-frequency components of the measurement, which therefore retains its validity. Moreover, limiting the analysis to the high-frequency band also rules out the effects of temperature variations, which occur, as the drift, only at frequencies of at most a few Hertz. This is the typical application scenario of distributed acoustic sensors (DAS), which can efficiently measure high-frequency strain variations. A rather effective strategy to track and compensate for the laser frequency drift in DAS consists in inserting a reference bobbin at the beginning of the fiber link [44]. The bobbin is kept in a stable environment; therefore, any variation recorded along the reference bobbin is ascribed to the laser drift and it is used to compensate for the rest of the measure. This approach lowers the minimum detectable acoustic frequency to the sub-Hertz range, but it still does not enable static monitoring.

The other application scenario is that of OFDR systems, where the laser source scans a rather wide bandwidth (typically from a few up to several tens of nanometers). This fact enables the use of reference gas cells [45], which provide a stable and absolute reference for the actual wavelength of the laser [46]. As a result, OFDR systems can efficiently monitor static and quasi-static variations of temperature and strain. This, however, leaves the temperature and strain variations indistinguishable. One approach to break this ambiguity is to use two fibers, one of which is not mechanically coupled to the structure being monitored and hence senses only its temperature variations [47]. This information is then used to correct the reading obtained from the other fiber, which is affected by both temperature and strain. Alternatively, a single fiber is used and the temperature is measured with Raman-based distributed temperature sensors, then the Rayleigh-based readout is corrected to obtain the strain as in the previous approach [48].

#### Strategies for Measuring Perturbations

As noted above, the sensitivity to temperature and strain variations occurs through the phase factor $e^{-j2\beta_{0}z}$; there are three main ways in which this fact can be exploited. The first one consists of launching a high-coherence pulse (without phase modulation) and detecting the backscattered power. Neglecting attenuation, the measured quantity is

(26) $$|b(z_{t}){|}^{2}={\left|\rho (z_{t})\right|}^{2}={\;\int \int \;}_{z_{t}-\Delta /2}^{z_{t}}\eta \left(z^{\prime }\right)\eta \left(z^{\prime \prime }\right)\cos\left(2\beta_{0}\left(z^{\prime }-z^{\prime \prime }\right)\right)dz^{\prime }dz^{\prime \prime }\;,$$

where we assumed for simplicity that the pulse is rectangular, and we exploited the fact that η is a real quantity. The resulting spatial resolution is equal to half the pulse length. Given its simplicity, this is the first kind of DAS ever proposed [2]; however, it suffers many drawbacks. The main ones are due to the fact that phase variations are mediated by the cosine function. Actually, because of this, the sensor response is neither linear nor monotonic, which makes the characterization of the acoustic field difficult if not impossible. Moreover, the cosine function has points of zero derivatives, which means that there are specific and unpredictable conditions in which the sensor sensitivity is null. Finally, owing to the random nature of η(z) there are points along the fiber where $\rho (z_{t})$ is close to zero, because of the destructive interference between the waves backscattered by the scattering centers illuminated by the pulse. These so-called *fading points* prevent the measurement and are quite detrimental; actually, removing the fading condition requires a very strong perturbation or a large variation of the laser frequency [2].

A better approach consists of measuring the phase of the backscattered light $b(z_{t})$. There are a few ways in which this can be achieved, exploiting either coherent or direct detection [2,49]. Nevertheless, whatever the approach, the measured quantity is (or is equivalent to) the interference between the light backscattered from two different positions along the fiber, namely $y\left(z_{t}\right)=b\left(z_{t}\right)b^{*}\left(z_{t}+L_{g}\right)$, where the *gauge length* $L_{g}$ sets the spatial resolution. In this case the measurement is mainly sensitive to phase variations occurring between $z_{t}$ and $z_{t}+L_{g}$, and since the complex quantity $y(z_{t})$ is measured, the DAS response is linear with respect to phase variations; this also implies that its sensitivity is constant. Nevertheless, this approach is also prone to fading points, just because when ρ approaches zero, so does *y*. Furthermore, the measurement is in this case also affected by *polarization fading*, which occurs when $b(z_{t})$ and $b^{*}\left(z_{t}+L_{g}\right)$ have close to orthogonal polarizations or when, in system with coherent receivers, $b(z_{t})$ is orthogonal to the local oscillator. Polarization fading can be avoided with polarization diversity receivers, at the expense of a more complex setup [2]. Finally, it must be noted that the phase is naturally defined between 0 and 2π, hence strong variations of temperature or strain can induce phase wrapping. Unwrapping algorithms can be used to restore the continuity of the measured phase, yet measurement noise can make the task daunting. Despite these difficulties, many commercial DAS systems are based on this approach.

Most of the issues listed above can be solved by exploiting the random nature of $\rho (z_{t})$, rather than measuring directly the phase of the backscattered light. This leads to a very effective sensing method based on the so-called *fiber fingerprint*. To better understand the method, it is convenient to resort to a discrete model of Rayleigh scattering, which assumes that the scattering originates from a series of randomly distributed discrete scattering centers. Mathematically, this is equivalent to assuming that

(27) $$\eta \left(z\right)=\sum_{k}\eta_{k}\delta \left(z-z_{k}\right)\;,$$

where $\eta_{k}$ and $z_{k}$ are the random intensity and position of the *k*th scattering center, the sum is extended to the scattering centers in the fiber section of interest, and δ(·) is the Dirac delta function. According to Equation (22) and neglecting attenuation and polarization effects, the round trip spectral response then becomes

(28) $$R_{1}\left(\Omega \right)=j\sum_{k}\eta_{k}\exp\left(-j\frac{2}{c_{0}}\left(\omega_{0}+\Omega \right)n_{0}z_{k}\right)\;,$$

where we set $\beta \left(\omega \right)=\omega n\left(\omega \right)/c_{0}$ and we approximated $n\left(\omega \right)\approx n_{0}$. The quantity $R_{1}\left(\Omega \right)$ is random; nevertheless, the quantities $n_{0}$, $z_{k}$ and $\omega =\omega_{0}+\Omega$ appear in the same product, suggesting the key idea behind the *spectral correlation analysis* of the Rayleigh fingerprint: the effects of a uniform relative variation δζ of the product $n_{0}z_{k}$ is equivalent to shifting the round trip spectral response by a proper amount Δω [14]. Specifically, the condition $\omega n_{0}z_{k}\left(1+\delta \zeta \right)=\left(\omega +\Delta \omega \right)n_{0}z_{k}$ yields the shift

(29) $$\Delta \omega =\omega \;\delta \zeta \approx \omega_{0}\;\delta \zeta \;.$$

This result shows that a uniform variation of temperature and/or strain along a fiber section, causes the corresponding spectral response to shift by a known amount; for single-mode silica fibers this shift is about 1.25 GHz/${}^{\circ }C$ and 0.15 GHz/μϵ [50,51]. The spectral shift is linearly dependent on the environmental variations and can be effectively measured by cross-correlating successive measurements. The range of measurable variations is limited by the measurement bandwidth, which is typically quite large in OFDR systems. Moreover, the method is not just insensitive to fading points, it actually exploits fading points, because as a matter of fact fading is what defines the fiber fingerprint, enabling the correlation analysis. As a result, this approach is successfully exploited in commercial OFDR systems to perform static distributed strain and temperature sensing.

More recently, the idea has been adapted to OTDR systems, too [52,53]. Actually, it has been shown that if the probe pulse is linearly chirped, any local variation of $n_{0}$ or $z_{k}$ leads to a time-shift of the backscattered trace equal to [52,54]

(30) $$\Delta t\approx \frac{\omega_{0}}{\sigma }\delta \zeta \;,$$

where σ is the chirp (frequency variation per unit of time) applied to the pulse. As before, the shift is linearly proportional to the variation and it can be detected by a cross-correlation analysis. The only drawback is that in order for the correlation to be robust, the bandwidths of the probe pulse and of the receiver have to be in the order of GHz, more than one order of magnitude larger than what is typical for standard OTDRs. This approach is known as chirped-pulse ϕ-OTDR [52] or time-gated OFDR [53] and it is at the base of extremely reliable DAS systems.

### 3.2. Polarization-Based Distributed Sensing

The idea of using optical fibers as distributed sensors was proposed for the first time by Alan J. Rogers in 1980, and it was based on the measurement of the polarization state of the Rayleigh-backscattered light [55,56]. Despite some intrinsic difficulties still hampering the full exploitation of the method, polarization-based distributed sensing has some unique abilities that keep the interest alive [57].

The state of polarization (SOP) of a light wave represented by the Jones vector v can be described by the associated *coherence matrix*, defined as $C_{v}=vv^{*}$; this quantity is totally equivalent to the Stokes vector (see Appendix C.5). Accordingly, and owing to (24), the SOP of the backscattered light is described by the coherence matrix

(31) $$C_{b}\left(z\right)=b\left(z\right)b^{*}\left(z\right)={\left|\rho (z)\right|}^{2}e^{-2\alpha (z)}V\left(z\right)C_{a}\left(z\right)V^{*}\left(z\right)\;,$$

where $C_{a}\left(z\right)$ is the coherence matrix—hence the SOP—of the forward propagating field $a\left(z\right)=U\left(z\right){\hat{a}}_{0}$ and, hereinafter, we use *z* instead of $z_{t}$ for simplicity. Note that $C_{b}$ is still affected by the random fading due to ρ(z); nevertheless, we can perform an average over a sliding window so that ${\left|\rho (z)\right|}^{2}$ is approximated by its mean value; then, neglecting also attenuation and after proper normalization, the round trip SOP can be expressed as

(32) $$C_{b}\left(z\right)=V\left(z\right)C_{a}\left(z\right)V^{*}\left(z\right)\;,$$

which is the main equation of distributed polarization measurements. The smoothing average of ${\left|\rho (z)\right|}^{2}$ is required to perform the analysis described below. In polarization-sensitive OTDR this averaging is mainly performed by the electrical receiver, whose bandwidth is typically fitted to the length of the probe pulse; a further contribution may come from the limited coherence of the laser source. Differently, in OFDR systems that have a spatial resolution in the order of millimeters or even tens of micrometers, the averaging must be performed numerically. In both cases, the averaging window should be shorter than the distance scale over which the backscattered SOP varies; at the same time, however, a short averaging window is less effective in smoothing the factor ${\left|\rho (z)\right|}^{2}$, leaving a more pronounced fading noise to impair the measurement. Note also that this smoothing average cannot be applied directly to b(z), because the average of ρ(z) is zero.

The quantity $C_{b}\left(z\right)$ is the raw data provided by polarization distributed measurement, and it represents the effects of the polarization properties of the fiber accumulated up to *z*. Yet, what we need to know for sensing applications are rather the local polarization properties. Retrieving this information requires solving an inverse scattering problem, which is sketched here and analyzed more in detail in Appendix C.5. As discussed in Section 2.4, the round trip response is invariant to a rotation of the reference frame around the fiber axis, and this fact can be used to separate the twist from the Faraday rotation. Accordingly, when referred to the rotated frame, the round trip SOP varies with *z* according to

(33) $$\partial_{z}C_{b}=-j2\left[K_{B}\left(z\right)C_{b}\left(z\right)-C_{b}\left(z\right)K_{B}\left(z\right)\right]\;,\;\mathrm{with}\;K_{B}\left(z\right)=V\left(z\right)\tilde{K}\left(z\right)V^{*}\left(z\right)\;,$$

where $\tilde{K}\left(z\right)$ is given by (17). Moreover, in the same reference frame the backward propagation matrix V(z) obeys the equation

(34) $$\partial_{z}V\left(z\right)=-jK_{B}\left(z\right)V\left(z\right)\;,\;V\left(0\right)=I\;.$$

We can now define the procedure to retrieve the local information. First of all, the backscattered SOP is measured to calculate the round trip coherence matrix $C_{b}\left(z\right)$; owing to (33) and repeating this measurement for different input SOPs, the round trip coupling matrix $K_{B}\left(z\right)$ can be calculated [35,58,59]. Once this quantity is known, Equation (34) can be solved, yielding the backward propagation matrix V(z). Finally, given V(z), the local polarization properties are determined by $\tilde{K}\left(z\right)=V^{*}\left(z\right)K_{B}\left(z\right)V\left(z\right)$.

Recalling Equation (17), we see that the imaginary part of $\tilde{K}\left(z\right)$ is related only to Faraday rotation. Remarkably, this quantity is independent of bending, twisting or any other perturbation that might affect the polarization of light. As reviewed in Section 4.6, this fact enables a rather reliable distributed measurement of the magnetic field parallel to the fiber axis, with an important application to the distributed monitoring of electric current. Differently, the real part of $\tilde{K}\left(z\right)$ depends on all the other possible perturbations; the effects of these perturbations are, in general, unpredictable except for twists. Actually, when the fiber is locally twisted, the matrix $\tilde{K}\left(z\right)$ undergoes a rotation proportional to the twist; therefore, by measuring this rotation, it is possible to monitor the local variations of the twist applied to the fiber (see Section 4.5).

While distributed twist sensing can be achieved by monitoring the strain along multiple fibers or along a multi-core fiber [60], polarization-based distributed sensing is the only technique able to achieve the same result using only one standard fiber. Even more remarkable is the ability of distributed magnetic field sensing, which is unparalleled by any other technique. Despite these unique features, polarization-based distributed sensing has not yet reached commercial maturity. There are two main issues hampering the wide adoption of the technique. One is the already mentioned residual fading noise. The other one is polarization mode dispersion, which is not accounted for in the model and tends to decrease the degree of polarization of the probing light, especially when small spatial resolution, hence large bandwidth is required. Both these effects limit the reliable applicability of distributed polarization sensing to distances of a few hundred meters.

## 4. Examples of Applications

In this section, we present some examples of the use of Rayleigh-based DOFSs to measure different parameters, including strain, pressure, vibration, temperature, twist, electric current and magnetic field. The scientific literature about these sensors is vast; therefore, we review a selected, yet not exhaustive, list of references and field of applications.

### 4.1. Distributed Strain Sensing

Distributed strain sensing finds applications in many diverse fields; here we focus mainly on the geophysical and geotechnical applications [61]. The features offered by Rayleigh-based sensing schemes, regarding the number of equivalent sensing points and measurement accuracy, befit the monitoring needs of many geophysical and geotechnical problems in static and dynamic regimes. Over the years, and especially after the commercialization of the first devices based on optical-frequency-domain reflectometry, different applications have been addressed, both in the laboratory and real-field.

In particular, the high spatial resolution attainable by the OFDR, associated with a reduced range, limited to some tens of meters, makes this technique suitable to be used in many small- and medium-scale physical models or devices to disclose new insights in the description of the monitored phenomena. For example, OFDR has been successfully applied to small-scale setups reproducing shallow landslide dynamics, demonstrating the capability of providing a detailed map of the strain field at the sliding surface with a sampling time adequate to detect the initiation of collapse [62,63]. The availability of compliant cables, with engineered sheaths, e.g., with ameliorated gripping, has been of paramount importance to guarantee an efficient coupling between the collapsing soil and the cable itself, investigated both theoretically [64,65,66,67] and experimentally, in soil [68] and concrete [69,70].

In particular, the effective integration of fiber optic sensors into concrete structures, along with the availability of proper cables, has allowed addressing an important issue regarding their integrity, i.e., the detection of cracks. Although some authors have addressed the problem using techniques such as FBGs [71,72,73] and Brillouin-based solutions [74,75,76], their coarse spatial resolution limits the capability of precisely locating the crack and quantitatively assessing its width. On the other hand, OFDR-based DOFS may guarantee a sufficient resolution, in the mm range or even below, which well befits the detection needs for this specific application. Casas and co-authors [77,78,79] have extensively investigated the feasibility of this approach, which has been further explored also by other groups [80,81,82]. In general, OFDR has been shown to be effective in detecting the position of multiple cracks, with an accuracy within 1 cm. Moreover, the accuracy in assessing the crack width is generally better for narrow cracks [81], yet is also affected by the specific cable structure [83].

**Figure 2:**
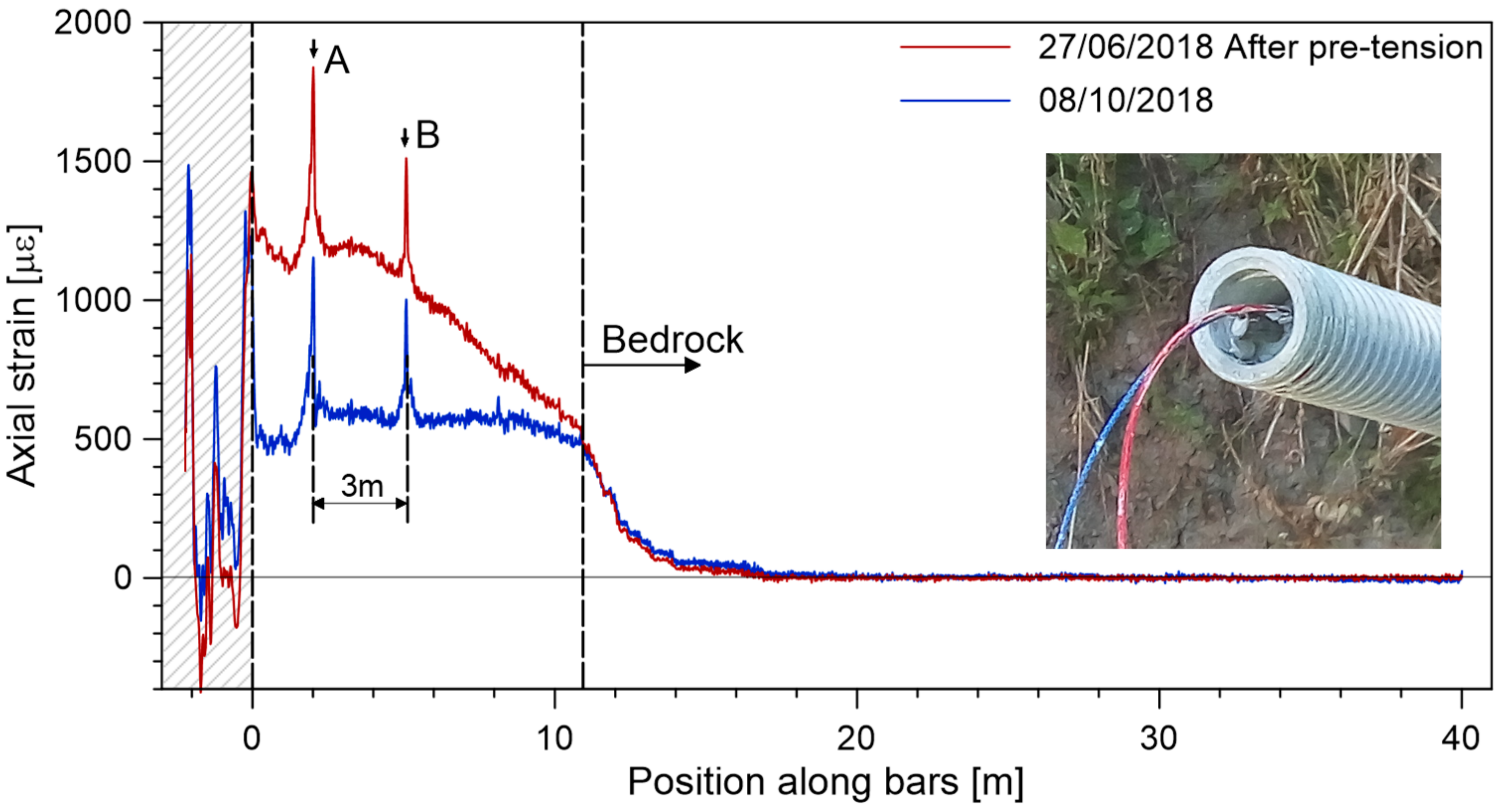
Axial strain along one soil anchor instrumented with a fiber optic and probed by a commercial OFDR system; the anchor was measured after some pre-tension was applied at the time of installation (blue curve) and then after about three months (red curve). In the inset, a picture of the head of a composite anchor equipped with fibers. The analysis of the strain profile shows that approximately one-quarter of the anchor length is activated up to the stable bedrock. Furthermore, the system’s high spatial resolution allows identification of the coupling nuts of the anchors, where peak strain values are recorded (marked by A, B), suggesting that those are points with lower stiffness (adapted from Ref. [84]).

Additional examples of the integration of DOFSs into the geotechnical structure, then interrogated with high-resolution OFDR, are represented by soil nails and foundation piles. Soil nails are steel bars inserted into the ground to remediate unstable slopes. They exert their retaining action by friction with the surrounding soil or rocks and anchoring to deeper and more stable soil or rock strata. The integration of DOFSs may be implemented either internally in hollow bars [84] or in a groove cut on the surface of the bars [85,86]. The fibers substantially allow measuring the strain exerted by the soil of the anchors. Much information about the health of the nail and the surrounding soil can be inferred from the strain analysis, including the nail length’s appropriateness, the remediation action’s effectiveness, and the evolution of the unstable slope. Figure 2 shows an example of the strain curve collected on the field by an anchor installed on an unstable slope. With a similar aim and methodology, optical fiber cables have also been integrated into foundation piles and then probed by an OFDR to monitor their strain under load [87,88,89].

In both the above applications, the required spatial resolution is generally in a few tens of centimeters. Although attainable by other distributed strain sensing techniques, such as those based on Brillouin optical time and frequency domain analysis, OFDR provides even higher spatial resolution by probing the cable from one single end, with evident practical advantage. Those alternative techniques, on the contrary, besides operating at the limit of their resolution, require accessing the fiber from both ends. Therefore, the cable has to be installed in a loop configuration, which may be complex for small-diameter devices such as nails and anchors.

### 4.2. Distributed Pressure Sensing

In general, the effect of pressure on an optical fiber consists of radial and longitudinal strain along with a change in the material density; the induced longitudinal strain is typically within the measurement range of the Rayleigh-based technique, enabling distributed pressure sensing. As a matter of fact, the pressure has been one of the parameters whose measurement was early envisaged using a phase-based Rayleigh technique [90,91], with a sensitivity of 0.1 MPa/με for a bare silica fiber.

The pressure sensitivity of a cabled or coated fiber can generally be tuned by compliant coatings, as extensively investigated by Lagakos and Bucaro in the 80’s [92]. Those studies were then extended to model acoustic sensitivity [93], since, from a mechanical point of view, the regime for which the pressure induces both radial and longitudinal strain extends to pressure waves up to 10 kHz [94]. Indeed, distributed pressure and acoustic sensing are firmly related, and are here separately addressed, only for the reader’s convenience.

In light of these considerations, it should not surprise the proposal to use a commercial Rayleigh-based fiber-optic distributed acoustic sensing system to measure pressure waves at a very low frequency (down to a few mHz) [95]. However, although this work has shown the approach’s feasibility, it required a significant elaboration effort on data. More recently, Mikhailov et al. [96] proposed the use of a highly birefringent photonic crystal fiber, probed by a φ-OTDR scheme to measure the differential pressure sensitivities between the slow and fast polarization axes, with a good sensitivity of 2000 MHz/MPa and a pressure uncertainty of 0.03 MPa; the measurement range was however short due to the large optical losses of the fiber. On the same track, the same group proposed a different microstructured fiber with low losses. They demonstrated the measurement over more than 700 m, with spatial resolution of 5 cm (i.e., resolving more than 14,000 sensing points), pressure sensitivity of 1590 MHz/MPa, and accuracy of 0.05 MPa [97]. Similarly, but in the time-domain, Gerosa et al. [98] probed a short embedded-core capillary fiber by a polarization-OFDR scheme showing a differential pressure sensitivity among polarization axes of approximately 500 MHz/MPa.

**Figure 3:**
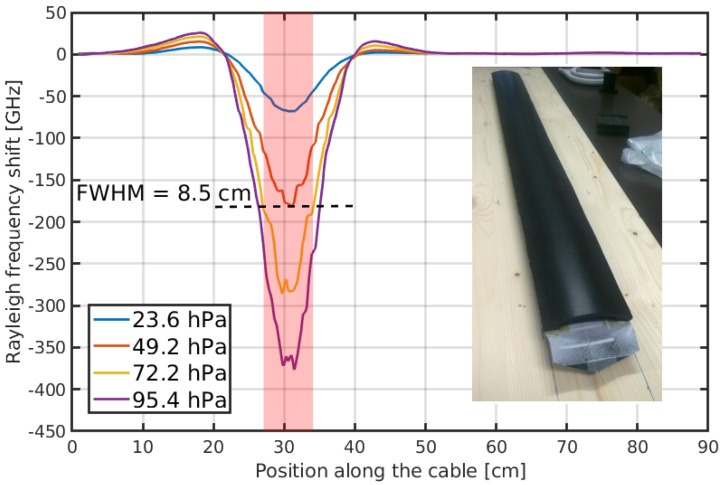
Response in terms of the Rayleigh frequency shift of the engineered cable proposed in [99] and shown in the inset as a function of the local pressure applied over the 7 cm-wide area around 30 cm. The corresponding full-width-half-maximum (FWHM) response extends up to 8.5 cm, showing a moderate non-local response of the cable to the pressure stimulus.

Promising as it is, the performance of these sensors is unfortunately not yet sufficient to measure pressure below some meters of equivalent water level, a range of interest for many important hydro-geological applications. The need for higher pressure sensitivity has pushed the community to focus on different approaches, such as the engineering of effective transducing structures embedding the fiber, which simultaneously improve the responsivity to pressure and allow distributed sensing. An example of such an approach is presented in Schenato et al. [99] in which a standard fiber was embedded in a zig-zag path within a compliant cable structure, made of two clamshell-like rubber profiles that push the fiber upon pressure. A 1 m-long prototype of this cable, interrogated by a commercial OFDR, showed a remarkable pressure sensitivity of 30 GHz/kPa with a spatial resolution of 8.5 cm, as shown in Figure 3. In this case, using a Rayleigh-based optical technique, such as OFDR, was not mandatory for regular operation. Nonetheless, it was of paramount importance for characterizing the inner working mechanism, given the high spatial resolution otherwise not attainable. For the same reason, other authors use OFDR for other quasi-distributed pressure sensors, which would be more easily implemented with other optical fiber sensors technology, such as FBGs [100,101,102].

### 4.3. Distributed Acoustic Sensing

Distributed acoustic sensing (DAS) is probably the most peculiar application of Rayleigh-based DOFSs. In these systems, the fiber is probed at a high frequency, enabling the distributed measurement of strain variation over relatively large bandwidths. Actually, the repetition rate is limited only by the round-trip time, so a 10 km long fiber can be probed at about 10 kHz, guaranteeing an acoustic bandwidth of about 5 kHz. DAS is sometimes referred to as distributed vibration sensor (DVS); some authors distinguish DAS from DVS on the bases that the DVSs can detect and locate the dynamic strain variation but cannot quantitatively measure it.

Nowadays, one of the fields of application in which DAS is gaining tremendous popularity is seismic monitoring; this is due to the outstanding spatial resolution, the low cost per single sensing point, and the possibility of using legacy fiber cable, originally installed for telecom applications. By employing a DAS system, an optical fiber can measure the surrounding vibration (i.e., the strain rate) with a resolution of a few meters over several kilometers as a very dense array of in-phase geophones [103]. Furthermore, the worst SNR of the data from the fiber with respect to standard geophones is compensated by a large number of sensing points and spatial coherency, allowing for the application of effective array processing techniques [104].

**Figure 4:**
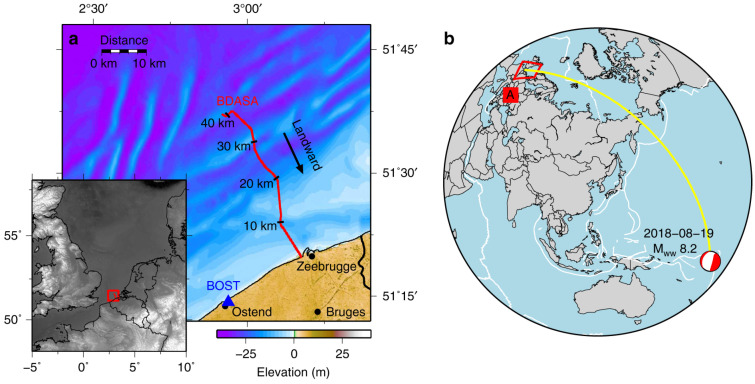
(**a**) The dark fiber cable (BDASA, Belgium Distributed Acoustic Sensing Array, red line) located at the Southern Bight of the North Sea offshore Zeebrugge, Belgium and used as a seismic array in [109]. (**b**) A world map showing the location of the BDASA (open red box, near the letter A), and the epicenter location (and Global Centroid Moment Tensor) of the 19 August 2018 Mw8.2 Fiji deep earthquake, which has been correctly detected by the DAS system. After [109].

Many works related to vertical seismic profiling have been published [105,106] and, more recently, many others about the use of DAS as a distributed seismic array for earthquake monitoring [107,108], showing the capability of detecting earthquakes thousands of kilometers far away from the fibers [109] (see Figure 4). One of the most foreseen features is the possibility of using already deployed dark fibers or unused channels of standard telecom fibers [108,109], with evident advantages of spatial coverage and minimal installation effort.

A recent, yet very promising, application of DAS as an ameliorate tool for seismic monitoring is detecting and tracking fast-moving landslides [110]. Initially demonstrated in a small-scale physical model of a landslide [111] and a debris flow [112], it has then been preliminarily validated in a large-scale experiment by Ravet et al. [113], by using explosive to emulate the short temporal and intense acoustic activity of rockfalls.

The works above represent a limited selection of the many other applications where DAS is currently being investigated. Almost daily, new areas of study, are being addressed: we briefly cite here structural health monitoring [114], road [115,116,117] and train [118,119] traffic, perimeter [120] and pipeline [121] patrolling, and even fauna [122,123] and insects [124] detection and tracking.

### 4.4. Temperature Sensing

Since the very beginning of DOFS, the temperature has been a parameter of great interest for the community of scientists working on Rayleigh-based distributed sensors [43,125,126,127,128]. Over the years, Raman and Brillouin scattering proved better approaches for standard application. In this regard, Rayleigh-based sensors only provide the temperature variations from a reference condition. At the same time, it is commonly assumed that Brillouin and Raman sensors allow for absolute temperature measurements, which may be desirable in some circumstances. However, only Raman scattering intrinsically provides the absolute temperature measurement encoded by the Stokes and Anti-Stokes signal intensity. On the contrary, the absolute temperature at any point along the fiber can be calculated from the Brillouin frequency shift only if the Brillouin spectrum is known along the fiber at a reference temperature [129]. In principle, the same approach may also work for Rayleigh-based sensors by implementing the absolute referencing of a first Rayleigh measurement, but, as far as we know, it has never been achieved. Nonetheless, Rayleigh-based techniques, primarily based on OFDR schemes, offer several key advantages in particular or exotic scenarios.

There are many applications in which the required spatial resolution is not attainable by other distributed temperature sensing techniques but Rayleigh ones. An example of this is represented by the work of Bersan et al., which used an OFDR to investigate the temperature variations induced by internal erosion on the temperature field in a small sandbox model, which required a centimetric spatial resolution [130].

The same need for high spatial resolution is also shared in other fields of application, as diverse as biomedical engineering [131,132] and monitoring of transformer cores [133,134], fuel-cells [135,136], or Li-ion batteries [137]. In biomedical engineering, the OFDR systems can become an important tool supporting medical treatments, where the precise spatial control of the temperature has to be assured not to harm the patient and, at the same time, to guarantee the effectiveness of the treatment. In monitoring electrical assets, high spatial resolution and small form factor are both essential to assess the health condition of devices without interfering with normal operation. This field of application further confirms the advantage of fiber optic sensors over standard ones regarding the immunity to electromagnetic fields, particularly in high-power electrical environments. In light of the transformation of the automotive industry toward electrification, we believe that fiber optic sensing technology will assume an even more relevant market position.

In a different, but still non conventional, scenario, Rizzolo and co-authors proposed OFDR-based monitoring of temperature in water pools for nuclear waste storage, showing the feasibility of the approach [138]. In the same years, the OFDR was demonstrated to be effective in detecting liquid sodium leakage from pipes of nuclear fast reactors [139,140]. In that specific application, Raman-based techniques are critical, given the differential radiation-induced attenuation for the Stokes and anti-Stokes.

**Figure 5:**
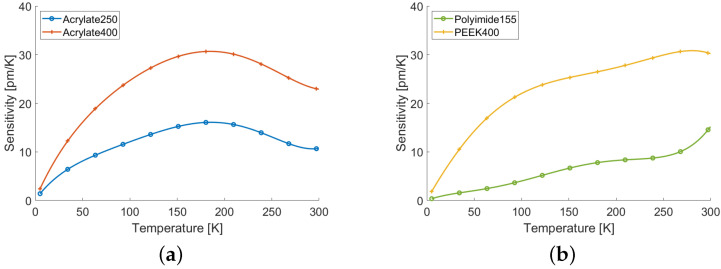
Temperature sensitivities in terms of the Rayleigh wavelength shift of SMF28 fibers with coatings of different material and thickness. The names in the legends indicate the coating material and the total diameter in micrometers of the coated fiber (adapted from Ref. [146]).

Rayleigh scattering has been proved to be effective in measuring temperature even at very high temperatures above 1000 K [141,142,143]. On the opposite extreme, Rayleigh scattering also offers the unique ability to perform distributed monitoring of cryogenic temperature, below about 50 K. Actually, at this low temperatures Raman and Brillouin cannot be used, because the former has no sensitivity, whereas the latter as a non-monotonic response [144]. Differently, Rayleigh retain sensitivity through the thermo-elastic effect of the coating applied to the fiber, which exerts strain on the silica in response to temperature variation [144,145]. Actually, by choosing the right material and, most importantly, thickness, it is possible to achieve different sensitivities [146]. For example, Figure 5 shows the temperature sensitivity due to different fiber coatings; the values refer to Rayleigh wavelength shift, and have been measured by a commercial OFDR. The results show the high nonlinearity of the response over the large temperature range, but, at the same time, they confirm that a proper coating can guarantee useful sensitivity even below 20 K.

### 4.5. Shape and Twist Sensing

A fascinating application of Rayleigh DOFSs is shape sensing [60]. The idea is that of measuring the path along which the fiber is deployed by measuring properties of the backscattered light. Typical applications are the measurements of shape profiles and the tracking of endoscopes. From the geometrical point of view, in order to achieve shape sensing it is necessary to measure the local curvature—both amplitude and direction—and twist along the fiber path; once these parameters are known as a function of the distance, the actual position in space of the fiber can be calculated [147].

In its most effective application, shape sensing exploits uncoupled multi-core fibers and measures the strain along each core [148,149,150]. By analyzing the differential strain between cores, it is possible to calculate the amplitude and direction of the local curvature. Differently, in order to also measure the twist, it is necessary to pre-twist the fiber, so to avoid ambiguity on the twist direction and minimize cross-sensitivity with temperature variation. In fact, temperature cross-sensitivity is mitigated just because the strain analysis is differential among cores, whereas temperature acts at the same way on each core.

The calculation of the absolute fiber position from the measurements of curvature and twist requires high accuracy and spatial resolution, in order to minimize error accumulation. For this reason, shape sensing is typically performed with OFDR and applied over distances of a few meters; in this condition, an uncertainty well below 1 mm on the position of the distal fiber end can be achieved [151].

**Figure 6:**
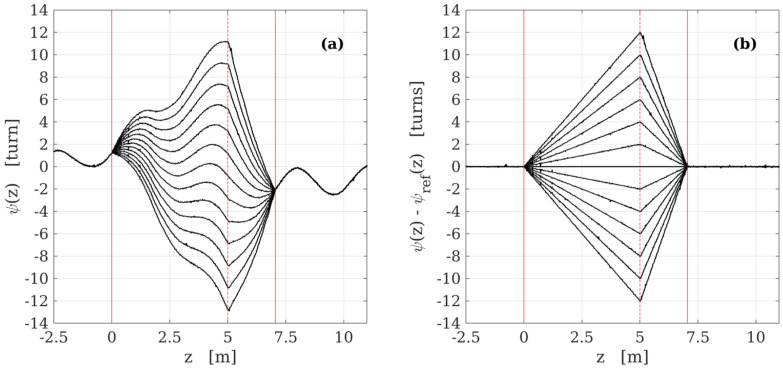
Polarimetric distributed measurement of twist along a single mode fiber for twists ranging from −12 to 12 turns, applied at the position marked by the dashed vertical line, while the fiber was fixed at positions marked by the solid vertical lines. (**a**) Raw angles of orientation of the linear birefringence vector; the undulatory pattern is due to intrinsic spin-induced rotation of fiber birefringence. (**b**) Orientation variations with respect to the reference measurement; the measurements accurately track the local twist applied to the fiber.

Another interesting example of geometrical sensing is twist monitoring [59], based on the measurement of the SOP of the Rayleigh backscattered field. As described in Section 3.2, twisting the fiber induces an apparent rotation of the local linear birefringence vector (in the Stokes space) equal to about 1.85 times the angle by which the fiber is rotated. This linear birefringence vector has its own orientation, which is typically random and has to be measured first as a reference. Once this is conducted, any local rotation applied to the fiber can be measured as the difference in orientation of the birefringence vector between the actual measurement and the reference one. Figure 6 shows an example of the result of this procedure. A fiber sample was laid straight fixed at two positions about 7.4 m apart and twisted in between by a known amount of turns; for each twist condition, the birefringence orientation was measured with a polarization-sensitive OFDR. Figure 6a shows the raw orientation of the linear birefringence vector; these angles account both for the applied twist and for the intrinsic birefringence orientation. Figure 6b shows the difference between these raw orientations and the reference one; now the data depend only on the twist and accurately agree with the local rotation applied to the fiber. The uncertainty in the twist measurement is typically in the order of a few degrees.

### 4.6. Distributed Sensing of Magnetic Field and Electric Current

Rayleigh-based DOFS can also be employed to perform distributed measurements of magnetic field and hence of electric current. There are two main approaches: the first one exploits special magnetostrictive coatings or structures that transduce magnetic field variations into strain variations, which are eventually measured with standard approaches [152]. While this method is quite effective in FBG point sensors, its application to distributed measurements is hampered by the difficulty of applying the specific coating on the whole fiber length.

Alternatively, distributed sensing of the magnetic field can exploit Faraday rotation, which occurs naturally in silica fibers and causes a nonreciprocal rotation of polarization proportional to the intensity of the magnetic field component parallel to the fiber axis [32,153]. As described in Section 3.2, due to its non-reciprocal nature, Faraday rotation has a unique effect on distributed polarization measurements that allows us to clearly distinguish it from other perturbations [36] (see also Appendix A.2).The only drawback of this approach is that the Verdet constant of silica, which quantifies its sensitivity to the magnetic field, is V≈0.6rad/(T·m) at 1550 nm. Such a low value limits the applicability of the approach only to rather high magnetic fields. For example, Ref. [57] describes the use of the technique to map the amplitude and direction of the magnetic field in the borehole of a 3 T MRI scanner, with a spatial resolution of a few centimeters and an accuracy of 100 mT.

**Figure 7:**
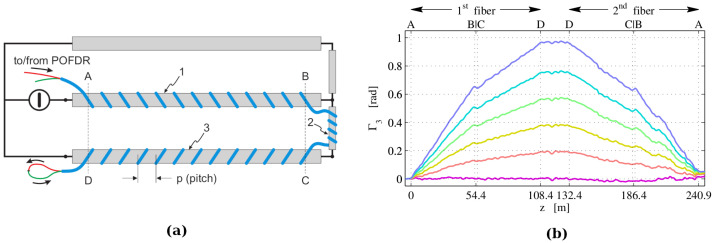
Polarimetric distributed measurement of electric current. (**a**) Sketch of the electrical circuit used to perform the experiment. The parallel conductors AB and CD have diameter 9 cm, are 20 m long and about 1.2 m apart; fibers are coiled with a pitch of 10 cm. (**b**) Faraday rotation measured along the concatenated fibers for current intensities ranging from 0 kA (lowest curve) to 2.5 kA (highest curve) in steps of 0.5 kA (Adapted from Ref. [154]).

The ability to measure magnetic fields leads naturally to the ability to measure electric currents, an application of great interest, especially in the perspective of monitoring high-energy electric links. The viability of the approach has been experimentally verified in Ref. [154] in a small-scale laboratory test. This consisted of the electrical circuit sketched in Figure 7a, with two fibers helically wound around the main conductors and connected at the distal end. The SOP of the backscattered light was measured with an OFDR for different intensities of current flowing in the circuit. It can be shown that in this configuration there is a simple relationship between the current intensity *I* flowing in the conductor and the accumulated Faraday rotation $\Gamma_{3}$ given by:

(35) $$\Gamma_{3}\left(z_{c}\right)=\frac{2\mu_{0}V}{p}\int_{0}^{z_{c}}s\left(z\right)I\left(z\right)dz\;,$$

where $z_{c}$ is the distance along the cable, *p* is the pitch of the fiber winding, and s(z)=±1 depending on whether the current is co- or counter-propagating with respect to the probe light, respectively.

Figure 7b shows the Faraday rotation accumulated along the fiber link, for different current intensities in the range 0– 2.5 kA. Some clear features confirm the soundness of the results; in particular, the fact that the slope in sections CD is about half that in sections AB is consistent with the fact that the current flowing in the latter is half that flowing in the former; moreover, the fact that the slopes are negative along the second fiber (which is traversed in the opposite direction) is consistent with the nonreciprocity of Faraday rotation. From these measurements, the currents could be measured with an accuracy of about 100 A and a spatial resolution of 4 m of electric cable.

## 5. Conclusions

In this paper, we have reviewed and commented on the theory behind Rayleigh-based distributed sensing and its application to several fields. Two main parts compose the paper: in the first one, we have presented a robust and complete theoretical model of Rayleigh scattering based on the formalism of scattering matrices. The model is rather general and also includes original results about multimode propagation and double scattering. In the effort of making the reading easier, many mathematical and implementation details have been deferred to an extensive Appendix for the readers’ reference.

The second part of the paper reviews selected and meaningful applications of Rayleigh-based DOFSs, considering strain, pressure, acoustic, temperature, shape and twist, and magnetic field and electric current sensing. For each measured physical parameter and specific application, we have discussed the advantages and features attainable by Rayleigh-based DOFS, along with the open issues and limitations. These are summarized in Table 1 with a (non-exhaustive) list of relevant references. In general, these technologies represent the only solution for those applications, which would otherwise not be possible to address due to the high spatial resolution and accuracy required. For that reason, these applications are often the most technically demanding implementations.

In general DOFS, including Rayleigh-based ones, are characterized by relevant costs and installation efforts. Nonetheless, when the application requires high spatial resolution and/or has to cover large areas, the advantages offered by DOFS in terms of general performance per sampling point are unmatched by any other technology.

## Figures and Tables

**Table 1:** Summary of the main Rayleigh-based DOFS applications reviewed in this paper.

Parameters (Technology)	Applications	Main Features	Open Issues	References
Strain (OFDR)	Small- and medium-scale physical models or devices; crack detection; geotechnical monitoring of anchors and piles	High sensitivity; ultra-high spatial resolution	Small distance range; temperature compensation	[61,62,63,64,65,66,67,68,69,70,71,72,73,74,75,76,77,78,79,80,81,82,83,84,85,86,87,88,89]
Pressure (OFDR)	General pressure measurements; water level monitoring	High spatial resolution	Limited sensitivity	[90,91,92,93,94,95,96,97,98,99,100,101,102]
Acoustic field (Φ-OTDR)	Seismic monitoring and VSP surveys; landslide and debris flows detecting and tracking; structural health monitoring; road and train traffic; perimeter and pipeline patrolling; fauna and insects detection	Long range; large number of sensing points; retrofitting of black fibers	Huge data storage; complex data analysis	[103,104,105,106,107,108,109,110,111,112,113,114,115,116,117,118,119,120,121,122,123,124]
Temperature (OFDR)	Small scale physical model; biomedical engineering; monitoring of transformer cores; fuel-cells or Li-ion batteries; application in harsh environments (high radiation and cryogenic environments)	High spatial resolution; extended temperature range	Relative measurement; small distance range	[43,125,126,127,128,130,131,132,133,134,135,136,137,138,139,140,141,142,143,144,145,146]
Shape and Twist (OFDR and POTDR)	Measurements of shape profiles; biomedical applications (endoscopes tracking)	High spatial resolution	Limited absolute position precision	[59,60,147,148,149,150,151]
Magnetic field and Electric current (POTDR)	MRI field characterization; high-energy cable current measurement	High spatial resolution; EMI-proof	Limited sensitivity	[32,36,57,152,153,154]

## Abbreviations

The following abbreviations are used in this manuscript:

DOAJ	Directory of open access journals
SNR	Signal-to-Noise-Ratio
OFS	Optical Fiber Sensor
DOFS	Distributed Optical Fiber Sensor
OTDR	Optical Time Domain Reflectometry
OFDR	Optical Frequency Domain Reflectometry
CW	Continuous Wave
CMT	Coupled-mode Theory
PDL	Polarization Dependent Loss
PMD	Polarization Mode Dispersion
DAS	Distributed Acoustic Sensing
DVS	Distributed Vibration Sensing
FBG	Fiber Bragg Grating

## Appendix A. Modelling of Optical Fibers by Coupled-Mode Theory

In this section, we review how Rayleigh scattering and fiber perturbations can be described by coupled-mode theory (CMT). In the CMT the coupling coefficients are expressed with respect to the modes of a reference fiber. We refer to the theory of Marcuse [28], which assumes that the reference fiber is reciprocal and lossless; note that the reference fiber has to be reciprocal, yet the perturbed one is not necessarily so. Let ${𝓔}_{\mu }^{\left(q\right)}$ and $H_{\mu }^{\left(q\right)}$ be the electric and magnetic fields of the mode μ of this reference fiber and let $\beta_{\mu }^{\left(q\right)}$ be the corresponding propagation constant; *q* can be either “+” or “−” to indicate that the mode is propagating forward or backward, respectively. The fact that the reference fiber is reciprocal allows us to conclude that [28]

(A1) $$\beta_{\mu }^{\left(-\right)}=-\beta_{\mu }^{\left(+\right)}\;,\;{𝓔}_{\mu }^{\left(-\right)}=M{𝓔}_{\mu }^{\left(+\right)}\;,\;H_{\mu }^{\left(-\right)}=-MH_{\mu }^{\left(+\right)}\;,\;M=\mathrm{diag}\left(1,\;1,\;-1\right),$$

where M is a diagonal matrix. Moreover, considering only guided modes, these must fulfill the following orthogonality condition

(A2) $${\;\int \int \;}_{S}\hat{z}\;\cdot \;\left({𝓔}_{\mu }^{\left(q\right)}\times {H_{\nu }^{\left(p\right)}}^{*}+{{𝓔}_{\nu }^{\left(p\right)}}^{*}\times H_{\mu }^{\left(q\right)}\right)dx_{t}=\left\{\begin{array}{cc} 4P\delta_{\mu ,\nu }\;, & \mathrm{for}\;q=p=\;“+”\\ -4P\delta_{\mu ,\nu }\;, & \mathrm{for}\;q=p=\;“-”\\ 0\;, & \mathrm{for}\;q\neq p \end{array}\right.$$

where *P* is an arbitrary normalization factor, $\delta_{\mu ,\nu }$ is the Kronecker delta and $x_{t}=\left(x_{1},\;x_{2}\right)$ is the vector of the transverse coordinate.

The CMT is an approximation; therefore, there are several slightly different ways to calculate the coupling coefficients. Following Marcuse’s approach and notation, the complex amplitude $c_{\mu }^{\left(q\right)}\left(z\right)$ of a generic mode μ propagating in direction *q* obeys the following differential equation

(A3) $$\partial_{z}c_{\mu }^{\left(q\right)}=-j\beta_{\mu }^{\left(q\right)}\;c_{\mu }^{\left(q\right)}\left(z\right)-j\sum_{\nu ,p}K_{\mu ,\nu }^{\left(q,p\right)}\left(z\right)c_{\nu }^{\left(p\right)}\left(z\right)\;,$$

where the coupling coefficients $K_{\mu ,\nu }^{\left(q,p\right)}\left(z\right)$ are given by

(A4) $$K_{\mu ,\nu }^{\left(q,p\right)}\left(z\right)=q\frac{\omega }{4P}{\;\int \int \;}_{S}{{𝓔}_{\mu }^{\left(q\right)}}^{*}\left(x_{t}\right)\;\Delta \varepsilon \left(x_{t},z\right)\;{𝓔}_{\nu }^{\left(p\right)}\left(x_{t}\right)dx_{t}\;,$$

where Δε is the (possibly anisotropic) dielectric perturbation applied to the reference fiber. With respect to the notation used in the main article, we see that $c_{\mu }^{\left(+\right)}\left(z\right)$ represents an element of the vector a(z), while $c_{\mu }^{\left(-\right)}\left(z\right)$ is an element of b(z). Similarly, comparing (A4) with Equation (8) of the main article, we have that

(A5) $$\begin{array}{ccc} \; & {\left\{K\right\}}_{\mu ,\nu }=K_{\mu ,\nu }^{\left(+,+\right)}\;,\;\; & {\left\{S_{1}\right\}}_{\mu ,\nu }=K_{\mu ,\nu }^{\left(-,+\right)}\;, \end{array}$$

(A6) $$\begin{array}{ccc} \; & {\left\{C\right\}}_{\mu ,\nu }=K_{\mu ,\nu }^{\left(-,-\right)}\;,\;\; & {\left\{S_{2}\right\}}_{\mu ,\nu }=K_{\mu ,\nu }^{\left(+,-\right)}\;, \end{array}$$

where ${\left\{A\right\}}_{\mu ,\nu }$ represents the element of position (μ,ν) of the matrix A. Similarly, $\beta_{\mu }^{\left(+\right)}$ is the element of position (μ,μ) of the diagonal matrix β.

The actual expressions of the coupling coefficients and hence of the coupling matrices depend on the specific perturbation Δε. Nevertheless, K and C are always real, except when the fiber is twisted or it is exposed to a strong magnetic field that causes Faraday rotation [33]. These two cases are important for sensing applications and are reviewed in the following for completeness. Moreover, the matrices $S_{1}$ and $S_{2}$ are mainly related to scattering or reflections, as discussed in Appendix A.3 and Appendix A.4.

### Appendix A.1. Fiber Twist

When the fiber is twisted by an angle ψ(z), two effects take place. The first one is of course the rotation of the fiber itself by the angle ψ(z). The second one is due to the shear stresses exerted on the silica and to the corresponding elasto-optical effect, which induces a perturbation of the permittivity tensor that reads [31,59]

(A7) $$\Delta \varepsilon_{\mathrm{twist}}=\epsilon_{0}n_{\mathrm{av}}^{2}\;g\;\tau \left(z\right)\left[\begin{array}{ccc} 0 & 0 & -x_{2}\\ 0 & 0 & x_{1}\\ -x_{2} & x_{1} & 0 \end{array}\right]\;,$$

where τ(z)=dψ/dz is the applied twist rate (rotation per unit length), g≈0.15 is the elasto-optic coefficient [59], and $n_{\mathrm{av}}$ is the mean refractive index. Note that this perturbation is the only one that couples transverse components of one mode with the longitudinal one of the other. Moreover, the fiber modes can be expressed in such a way that the transverse components are real, while the longitudinal ones are purely imaginary [155]. As consequence, the coupling coefficients $K_{\mu ,\nu }^{\left(q,p\right)}\left(z\right)$ given by (A4) are imaginary. It is worthwhile remarking that twist, along with Faraday rotation discussed in the next section, is the only perturbation that causes an imaginary coupling matrix; in all the other cases K(z) is real. We also note that the sign of the perturbation does not depend on the direction of propagation of light, as it happens for all other perturbations, except Faraday rotation. This confirms that the twist is a reciprocal perturbation. Finally, note that the twist does not induce losses, therefore the corresponding coupling matrix is Hermitian and it can be written as $K=jK_{\mathrm{twist}}$, where $K_{\mathrm{twist}}$ is real and anti-symmetric.

### Appendix A.2. Faraday Rotation

A magnetic field with a strong component parallel to the fiber axis induces a rotation of polarization known as Faraday rotation [32]. The phenomenon is described by the following perturbation of the permittivity tensor:

(A8) $$\Delta \varepsilon_{\mathrm{faraday}}=j\frac{\lambda n_{a}v\epsilon_{0}}{\pi }VB\cos\phi \left[\begin{array}{ccc} 0 & -1 & 0\\ 1 & 0 & 0\\ 0 & 0 & 0 \end{array}\right]\;,$$

where λ is the wavelength, *B* is the magnetic flux density, ϕ is the angle subtended by the magnetic field and the *direction of propagation* of light, and *V* is the Verdet constant, which is about 0.6 rad/(T · m) in silica optical fibers at 1550 nm [36].

Similarly to twist but for a totally different reason, Faraday rotation also causes imaginary coupling coefficients. Most important and in contrast with all other kinds of perturbation, the sign of the Faraday rotation depends on the direction of propagation of light through the angle ϕ, confirming that this phenomenon is nonreciprocal. Faraday rotation does not induce losses, therefore its coupling matrix for forward propagating modes is Hermitian and it can be written as $K=jK_{\mathrm{faraday}}$, where $K_{\mathrm{faraday}}$ is real and anti-symmetric. The same can be said about the coupling matrix for the backward propagating modes $C=jC_{\mathrm{faraday}}$. Moreover, as mentioned above, the only effect of changing the direction of propagation is a change of the sign of the perturbation; therefore, given that in reciprocal fiber we have $C=-K^{T}$, in this nonreciprocal case it must be $C=K^{T}$, and hence $C_{\mathrm{faraday}}=K_{\mathrm{faraday}}^{T}=-K_{\mathrm{faraday}}$.

### Appendix A.3. Rayleigh Scattering

The above framework can be used to calculate the contribution of Rayleigh scattering to the matrices $S_{1}$ and $S_{2}$. The origin of Rayleigh scattering can be ascribed to small random fluctuations of the dielectric permittivity on a sub-wavelength spatial scale [37]. In general these fluctuations may be slightly anisotropic [2]; nonetheless, to the aim of this model we can safely assume them scalar. Therefore, Rayleigh scattering is here described by a *scalar and real* random spatial fluctuation of the dielectric constant, $\Delta \varepsilon (x_{t},z)$, which allows us to calculate the induced coupling coefficients as

(A9) $$K_{\mu ,\nu }^{\left(q,p\right)}\left(z\right)=q\frac{\omega }{4P}{\;\int \int \;}_{S}\Delta \varepsilon \left(x_{t},z\right){{𝓔}_{\mu }^{\left(q\right)}}^{*}\left(x_{t}\right){𝓔}_{\nu }^{\left(p\right)}\left(x_{t}\right)dx_{t}\;.$$

This expression shows that the contribution of Rayleigh scattering depends on the “degree of overlap” of the involved modes. Being Δε a random process, it is possible to make a statistical analysis of these coupling coefficients. In particular, we assume that Δε is a random spatial process with zero average and a given spatial correlation, as

(A10) $$\begin{array}{cc} \langle \Delta \varepsilon (x)\rangle & =0\;, \end{array}$$

(A11) $$\begin{array}{cc} \langle \Delta \varepsilon \left(x\right)\Delta \varepsilon \left(x^{\prime }\right)\rangle & =r(x-x^{\prime })\;. \end{array}$$

where we have introduced the coordinate $x=\left(x_{t}\;,z\right)=\left(x_{1},\;x_{2},\;z\right)$ and the generic auto-correlation function *r*, and we have assumed, as somewhat reasonable, that Δε is a stationary random process [37]. From these assumptions, it is straightforward to verify that

(A12) $$\langle K_{\mu ,\nu }^{\left(q,p\right)}\rangle =q\frac{\omega }{4P}{\;\int \int \;}_{S}\langle \Delta \varepsilon (z)\rangle {𝓔}_{\mu }^{*}{𝓔}_{\nu }dx_{t}=0\;;$$

so that the mean value of $S_{1}$ and $S_{2}$ is zero. Differently, the evaluation of the second order statistical momenta is more cumbersome; as an example, the correlation of $K_{\mu ,\nu }^{\left(q,p\right)}$ is

(A13) $$\langle K_{\mu ,\nu }^{\left(q,p\right)}\left(z\right){K_{\mu ,\nu }^{\left(q,p\right)}}^{*}\left(z^{\prime }\right)\rangle ={\left(\frac{\omega }{4P}\right)}^{2}{\;\int \int \;}_{S}{\;\int \int \;}_{S}r\left(x-x^{\prime }\right)\;{{𝓔}_{\mu }^{\left(q\right)}}^{*}\left(x_{t}\right){𝓔}_{\nu }^{\left(p\right)}\left(x_{t}\right){𝓔}_{\mu }^{\left(q\right)}\left(x_{t}^{\prime }\right){{𝓔}_{\nu }^{\left(p\right)}}^{*}\left(x_{t}^{\prime }\right)\;dx_{t}\;dx_{t}^{\prime }\;.$$

To evaluate this integral, we have to make some assumptions on the correlation $r(x-x^{\prime })$. In particular, we assume that the correlation length of Δε is (in any direction) much shorter than the distance over which the field distribution varies [37]; accordingly, we approximate the correlation function to a Dirac delta as

(A14) $$r\left(x\right)\approx \sigma^{2}\delta \left(x\right)=\sigma^{2}\delta \left(x_{1}\right)\delta \left(x_{2}\right)\delta \left(z\right)\;,$$

where σ is the standard deviation of the fluctuation Δε. Now, using the sampling property of the Dirac delta function we can write

(A15) $$\langle K_{\mu ,\nu }^{\left(q,p\right)}\left(z\right){K_{\mu ,\nu }^{\left(q,p\right)}}^{*}\left(z^{\prime }\right)\rangle ={\left(\frac{\omega \sigma }{4P}\right)}^{2}\;\delta \left(z-z^{\prime }\right){\;\int \int \;}_{S}\;{\left|{{𝓔}_{\mu }^{\left(q\right)}}^{*}\;{𝓔}_{\nu }^{\left(p\right)}\right|}^{2}dx_{t}\;.$$

This analysis shows that the coefficients $K_{\mu ,\nu }^{\left(q,p\right)}\left(z\right)$, hence the elements of $S_{1}$ and $S_{2}$, are zero-average, δ-correlated random processes. Note also that (A15) is never zero except for orthogonally polarized modes, so Rayleigh scattering couples each mode to any co-polarized one; the intensity of this coupling depends however on the overlap integrals, some of which may indeed be close to zero [38].

We now specialize the analysis to the scattering matrix $S_{1}$; similar conclusions hold for $S_{2}$. Owing to (A1) we have

(A16) $${\left\{S_{1}\right\}}_{\mu ,\nu }=K_{\mu ,\nu }^{\left(-,+\right)}\left(z\right)=-\frac{\omega }{4P}{\;\int \int \;}_{S}\Delta \varepsilon \left(z\right){{𝓔}_{\mu }^{\left(+\right)}}^{*}M{𝓔}_{\nu }^{\left(+\right)}dx_{t}\;.$$

In weakly guiding fibers the matrix M plays a marginal role, because it just changes sign to the longitudinal components, which are negligible; as a consequence, the correlations of the coupling coefficients read

(A17) $$\begin{array}{cc} \langle K_{\mu ,\mu }^{\left(-,+\right)}\left(z\right){K_{\mu ,\mu }^{\left(-,+\right)}}^{*}\left(z^{\prime }\right)\rangle & \approx {\left(\frac{\omega \sigma }{4P}\right)}^{2}\;\delta \left(z-z^{\prime }\right){\;\int \int \;}_{S}\;{\left|{𝓔}_{\mu ,t}^{\left(+\right)}\right|}^{4}dx_{t} \end{array}$$

(A18) $$\begin{array}{cc} \langle K_{\mu ,\nu }^{\left(-,+\right)}\left(z\right){K_{\mu ,\nu }^{\left(-,+\right)}}^{*}\left(z^{\prime }\right)\rangle & \approx {\left(\frac{\omega \sigma }{4P}\right)}^{2}\;\delta \left(z-z^{\prime }\right){\;\int \int \;}_{S}\;{\left|{{𝓔}_{\mu ,t}^{\left(+\right)}}^{*}\;{𝓔}_{\nu ,t}^{\left(+\right)}\right|}^{2}dx_{t} \end{array}$$

whereas their cross-correlations are

(A19) $$\begin{array}{cc} \langle K_{\mu ,\nu }^{\left(-,+\right)}\left(z\right){K_{\eta ,\xi }^{\left(-,+\right)}}^{*}\left(z^{\prime }\right)\rangle & \approx {\left(\frac{\omega \sigma }{4P}\right)}^{2}\;\delta \left(z-z^{\prime }\right){\;\int \int \;}_{S}\left({{𝓔}_{\mu ,t}^{\left(+\right)}}^{*}{𝓔}_{\nu ,t}^{\left(+\right)}\right)\left({{𝓔}_{\xi ,t}^{\left(+\right)}}^{*}{𝓔}_{\eta ,t}^{\left(+\right)}\right)\;dx_{t}^{\prime } \end{array}$$

(A20) $$\begin{array}{cc} \langle K_{\mu ,\nu }^{\left(-,+\right)}\left(z\right)K_{\eta ,\xi }^{\left(-,+\right)}\left(z^{\prime }\right)\rangle & \approx {\left(\frac{\omega \sigma }{4P}\right)}^{2}\;\delta \left(z-z^{\prime }\right){\;\int \int \;}_{S}\left({{𝓔}_{\mu ,t}^{\left(+\right)}}^{*}{𝓔}_{\nu ,t}^{\left(+\right)}\right)\left({{𝓔}_{\eta ,t}^{\left(+\right)}}^{*}{𝓔}_{\xi ,t}^{\left(+\right)}\right)\;dx_{t}^{\prime }\;. \end{array}$$

To summarize, the elements of the Rayleigh scattering matrix $S_{1}$ are zero-mean, δ-correlated random processes. The correlations and cross-correlations of these elements depend on specific overlap integrals between the involved modes. In general, we should expect that the elements on the matrix diagonal should be those with the largest rms value, while the off-diagonal elements should have smaller rms values; nevertheless, these are not zero unless they relate to orthogonally polarized modes.

### Appendix A.4. About the Interpretation of the Scattering Matrices

So far, we have attributed to the matrices $S_{1}$ and $S_{2}$ the role of describing Rayleigh scattering. Nonetheless, according to the general expression (A4) of the coupling coefficients, also any other perturbation such as twist, bending, etc., can contribute to those matrices. Here we show why these contributions are largely negligible.

We introduce a transformation to compensate for the phase delays due to the propagation constants; namely, we define the complex amplitudes

(A21) $$\left[\begin{array}{c} \tilde{a}\left(z\right)\\ \tilde{b}\left(z\right) \end{array}\right]=\left[\begin{array}{cc} \exp(j\beta z) & 0\\ 0 & \exp(-j\beta z) \end{array}\right]\left[\begin{array}{c} a(z)\\ b(z) \end{array}\right]\;.$$

As shown in Appendix C.4, the round-trip spectral response $R_{1}\left(L\right)$ is invariant to the above transformation and can be expressed as

(A22) $$R_{1}\left(L\right)=j\int_{0}^{L}\tilde{B}\left(e^{-j\beta z}S_{1}e^{-j\beta z}\right)\tilde{F}dz\;,$$

where, neglecting double scattering for simplicity, the transformed forward and backward matrix responses obey the equations

(A23) $$\partial_{z}\tilde{F}=-j\left(e^{j\beta z}K\left(z\right)e^{-j\beta z}\right)\tilde{F}\;,\;\;\partial_{z}\tilde{B}=j\tilde{B}\left(e^{j\beta z}C\left(z\right)e^{-j\beta z}\right)\;.$$

Note that the elements of exp(−jβz) vary on the scale of the modes’ effective wavelengths, which is in the order of about 1 μm. However, in (A23) these elements are always multiplied by their complex conjugates; therefore, they give rise to factors varying on the scale of the modal beat lengths, which are of orders ranging from tens of micrometers, in multimode fibers, to several meters, in single-mode fibers. Similarly, the perturbations and hence the matrices K(z) and C(z) also typically vary on scales longer than centimeters. As a result, both $\tilde{F}\left(z\right)$ and $\tilde{B}\left(z\right)$ vary on scales longer than tens of micrometers—or even longer than centimeters in single-mode fibers.

The elements of exp(−jβz) appear also in the expression of $R_{1}\left(L\right)$, (A22); this time, however, they are multiplied by themselves giving rise to terms such as

(A24) $$\int_{0}^{L}f\left(z\right)\exp\left(-j\left(\beta_{\mu }+\beta_{\nu }\right)z\right)dz\;,$$

where the function f(z) is equal to a proper combination of the elements of $\tilde{F}$, $\tilde{B}$ and $S_{1}$. Note that (A24) is mathematically equivalent to a Fourier transform evaluated at the rather high spatial frequency $\beta_{\mu }+\beta_{\nu }$, which corresponds to a spatial scale shorter than the effective wavelength. Clearly, this kind of term can give a non-negligible contribution only if f(z) has non-negligible variations on a comparable length scale. Nevertheless, according to the above argument, this contribution cannot come from $\tilde{F}$ or $\tilde{B}$, neither it can come from $S_{1}$, as long as it describes slowly varying perturbations such as bending, twist, etc. Differently, as discussed in Appendix A.3, when $S_{1}$ describes the coupling due to Rayleigh scattering it varies on a very short scale, yielding the only non-negligible contribution to $R_{1}\left(L\right)$. For this reason, the only significant role of $S_{1}\left(z\right)$ is to describe Rayleigh scattering; of course, a similar argument applies to $S_{2}\left(z\right)$.

We conclude by remarking that a noticeable exception to the above analysis is when the fiber is purposely perturbed on a sub-wavelength scale, such as in fiber Bragg gratings. This case is however out of the scope of the present analysis.

## Appendix B. Numerical Solution of the Propagation Equations

The calculation of the fields transmitted and scattered across the fiber requires, in the most general case, the solution of the Riccati matrix Equation (12). Notice that the equation is nonlinear; however, it is relatively easy to verify by direct differentiation that its solution can be factorized as

(A25) $$R_{2}\left(z\right)=X\left(z\right)Y^{-\;1}\left(z\right)\;,$$

where the matrices X and Y are the solutions of the linear matrix equation

(A26) $$\left[\begin{array}{c} \partial_{z}X\\ \partial_{z}Y \end{array}\right]=-j\left[\begin{array}{cc} Q & S_{2}\\ S_{1} & H \end{array}\right]\left[\begin{array}{c} X\\ Y \end{array}\right]\;,\;\mathrm{with}\;X\left(0\right)=Y\left(0\right)=I\;;$$

We remark the analogy between (8) and (A26) of the main article. Equation (A26) can be numerically solved with the standard approach based on the waveplate model. Once $R_{2}\left(z\right)$ has been calculated, F and B can be determined solving (10) and (11); this can be performed numerically resorting again to the waveplate model.

An alternative approach consists in solving the matrix differential equation

(A27) $$\partial_{z}T=-j\left[\begin{array}{cc} Q & S_{2}\\ S_{1} & H \end{array}\right]T\;,\;T\left(0\right)=I\;,$$

where T is the 2N×2N complex matrix such that

(A28) $$\left[\begin{array}{c} a(z)\\ b(z) \end{array}\right]=T\left(z\right)\left[\begin{array}{c} a(0)\\ b(0) \end{array}\right]=\left[\begin{array}{cc} T_{1,1}\left(z\right) & T_{1,2}\left(z\right)\\ T_{2,1}\left(z\right) & T_{2,2}\left(z\right) \end{array}\right]\left[\begin{array}{c} a(0)\\ b(0) \end{array}\right]\;.$$

Then, comparing (5) with (A28) we reach the following relationships:

(A29) $$\begin{array}{cccc} & F=T_{1,1}-T_{1,2}T_{2,2}^{-\;1}T_{2,1}\; & & R_{2}=T_{1,2}T_{2,2}^{-\;1}\\ & R_{1}=-T_{2,2}^{-\;1}T_{2,1} & & B=T_{2,2}^{-\;1}\;. \end{array}$$

Similarly to the other approach, (A27) can also be solved using the waveplate model.

Considering the generic equation $\partial_{z}A=W\left(z\right)A\left(z\right)$, the waveplate model approach consists in dividing the integration domain in waveplates of length $L_{w}$ short enough to assume that W(z) is constant across the waveplate. Letting $W_{n}$ be the constant value of W(z) across the *n*th waveplate, the solution at each step is determined analytically as $A_{n+1}=\exp\left(W_{n}L_{w}\right)A_{n}$. There are several ways to compute the matrix exponential [156], yet all of them have complexity proportional to the cube of the matrix dimension. Accordingly, the complexity of solving (A27) is proportional to $8N^{3}$, to which we should add the cost of calculating the inverse of matrix $T_{2,2}$ in (A29); nonetheless, this need not be calculated when the fiber is reciprocal.

## Appendix C. Mathematical Proofs

### Appendix C.1. Proof of Equation (6) of the Main Article

Before taking the *z*-derivative of (5), note that a(0) is independent of *z* trivially because it is the boundary value that we impose on the problem (i.e., the way in which light is launched in the fiber). More subtly, b(0) is also independent of *z*, but for a completely different reason; actually, b(0) is independent of *z* because *z* is just an observation point. The value of b(0) is given by (2) along with the boundary values a(0) and b(L); as long as the fiber length *L* does not change (and it does not change in this model), b(0) is constant, regardless of the point *z* where we observe the field. In other words, it should be kept in mind that changing *z* does not mean changing the length of the fiber, which is fixed at *L*. Owing to the above argument, the *z*-derivative of (5) read

(A30) $$\left[\begin{array}{c} \partial_{z}a\left(z\right)\\ \partial_{z}b\left(z\right) \end{array}\right]=\left[\begin{array}{cc} \partial_{z}F-\partial_{z}\left(R_{2}B^{-\;1}R_{1}\right) & \partial_{z}\left(R_{2}B^{-\;1}\right)\\ -\partial_{z}\left(B^{-\;1}R_{1}\right) & \partial_{z}B^{-\;1} \end{array}\right]\left[\begin{array}{c} a(0)\\ b(0) \end{array}\right]\;.$$

Starting from Equation (2), it is possible to prove by standard algebraic manipulation that

(A31) $$\left[\begin{array}{c} a(0)\\ b(0) \end{array}\right]=\left[\begin{array}{cc} F^{-\;1} & -F^{-\;1}R_{2}\\ R_{1}F^{-\;1} & B-R_{1}F^{-\;1}R_{2} \end{array}\right]\left[\begin{array}{c} a(z)\\ b(z) \end{array}\right]\;,$$

which is the inverse relation of (5). Inserting (A31) in (A30) and reordering the terms with the help of the property $\partial_{z}G^{-\;1}=-G^{-\;1}\left(\partial_{z}G\right)G^{-\;1}$, we finally reach (6).

### Appendix C.2. Proof of Equation (13) of the Main Article

As recalled in Equation (3) of the main article, reciprocity implies that $R_{1}\left(z\right)$ and $R_{2}\left(z\right)$ must be symmetric and that $B^{T}\left(z\right)=F\left(z\right)$. Given these relationships, using (9) we obtain

(A32) $$R_{1}\left(L\right)=j\int_{0}^{L}F^{T}\left(z\right)S_{1}\left(z\right)F\left(z\right)dz=j\int_{0}^{L}F^{T}\left(z\right)S_{1}^{T}\left(z\right)F\left(z\right)dz=R_{1}^{T}\left(L\right)\;.$$

In this expression, the integration extrema 0 and *L* are totally arbitrary and refer to the section of fiber being “cut” out of a longer fiber; given that reciprocity holds for every fiber section, we must conclude that $S_{1}\left(z\right)=S_{1}^{T}\left(z\right)$. We now use (10) and (11) to evaluate $\partial_{z}B^{T}=\partial_{z}F$ and, recalling that $R_{2}=R_{2}^{T}$, we find

(A33) $$Q+H^{T}=R_{2}S_{1}-R_{2}^{T}S_{1}^{T}=0\;,$$

hence $H=-Q^{T}$. Inserting these results in (12) we reach

(A34) $$\partial_{z}R_{2}=-j\left(Q\;R_{2}+R_{2}Q^{T}-R_{2}S_{1}R_{2}+S_{2}\right)\;,\;R_{2}\left(0\right)=I\;,$$

which also implies that $S_{2}=S_{2}^{T}$, because $R_{2}$ is symmetric. So we can prove Equation (13).

### Appendix C.3. Proof of Equation (14) of the Main Article

A generic device is lossless if and only if its scattering matrix S is unitary, that is $S^{-\;1}=S^{*}$ [25]. Consider now a lossless fiber; any arbitrary subsection comprised between 0 and a generic point *z* can be described by a unitary scattering matrix S(z). Omitting the dependence on *z* for compactness of notation, since $S^{T}S=SS^{T}=I$, it is easy to verify by direct calculation that the following equations must hold for every *z*

(A35) $$\begin{array}{cc} \; & BB^{*}+R_{1}R_{1}^{*}=B^{*}B+R_{2}^{*}R_{2}=I\;, \end{array}$$

(A36) $$\begin{array}{cc} \; & FF^{*}+R_{2}R_{2}^{*}=F^{*}F+R_{1}^{*}R_{1}=I\;, \end{array}$$

(A37) $$\begin{array}{cc} \; & B^{*}R_{1}=-R_{2}^{*}F\;, \end{array}$$

(A38) $$\begin{array}{cc} \; & BR_{2}^{*}=-R_{1}F^{*}\;. \end{array}$$

Taking the *z*-derivative of (A37), using Equations (9)–(12) and (A35), we reach the following result

(A39) $$S_{1}+S_{2}^{*}=R_{2}^{*}\left(Q-Q^{*}\right)\;;$$

similarly, starting from (A38) and using (A36) we find

(A40) $$S_{1}+S_{2}^{*}=\left(H^{*}-H\right)R_{2}^{*}\;.$$

These equations must be verified for every *z*; moreover, all the matrices describe a local property except $R_{2}$, which describes a property accumulated along the fiber. It is therefore reasonable to conclude that those equations can be verified for every *z* if and only if $Q^{*}=Q$, $H=H^{*}$, and $S_{2}=-S_{1}^{*}$. Alternatively, we can note that for *z* approaching 0, $R_{2}\left(z\right)$ tends to zero, according to (12); this yields $S_{2}\left(0\right)=-S_{1}^{*}\left(0\right)$. Nevertheless, there is nothing special in z=0, which is just an arbitrarily chosen reference point; as a consequence, $S_{1}+S_{2}^{*}$ must be 0 for every *z*, hence we can also conclude that $Q^{*}=Q$ and $H=H^{*}$.

It is worthwhile remarking that assuming S unitary does not mean that the fiber has no transmission losses. In fact, it means that all the injected power exits from the fiber, part by transmission and part by reflection. Assuming no transmission losses means that F(z) is unitary; then, owing to (A35) and (A36), we can easily conclude that in this case B(z) is also unitary, while $R_{1}\left(z\right)=R_{2}\left(z\right)=0$. Clearly, if all the power is transmitted, no power can be reflected. Another interesting case is that of fiber with no mode-dependent loss (MDL). No MDL means that the forward propagation matrix can be factorized as $F\left(z\right)=\alpha \left(z\right)F_{U}\left(z\right)$, where α(z) is real and accounts for field attenuation and $F_{U}\left(z\right)$ is unitary. Under this assumption, Equations (A35) and (A36) imply that the same factorization can be made also for the other matrices; more specifically, we find $B=\alpha B_{U}$, $R_{1}=\rho R_{1U}$ and $R_{2}=\rho R_{2U}$, with $\rho^{2}=1-\alpha^{2}$. Note, however, that owing to Equation (10) of the main article, the assumption $F\left(z\right)=\alpha \left(z\right)F_{U}\left(z\right)$ put constraints on Q, $S_{1}$ and $R_{2}$ that do not seems physically possible. Yet these constraints drop when double scattering can be neglected. In this case, having $F\left(z\right)=\alpha \left(z\right)F_{U}\left(z\right)$ requires, as known, that Q(z) is such that Q−tr(Q) is Hermitian, with tr(·) the trace of the matrix.

### Appendix C.4. Proof of the Invariance with Respect to Rotation of the Reference Frame

We prove here that the round-trip spectral response $R_{1}\left(L\right)$ is invariant to a rather large class of transformations. Specifically, we transform the vectors of complex amplitudes according to

(A41) $$\left[\begin{array}{c} \tilde{a}\left(z\right)\\ \tilde{b}\left(z\right) \end{array}\right]=\left[\begin{array}{cc} T_{1}\left(z\right) & 0\\ 0 & T_{2}\left(z\right) \end{array}\right]\left[\begin{array}{c} a(z)\\ b(z) \end{array}\right]\;,$$

where $T_{1}\left(z\right)$ and $T_{2}\left(z\right)$ are arbitrary complex transformations with the only constraint of being invertible and such that $T_{1}\left(0\right)=T_{2}\left(0\right)=I$. Owing to Equation (2) of the main article we can write

(A42) $$\begin{array}{c} \left[\begin{array}{c} b(0)\\ \tilde{a}\left(L\right) \end{array}\right]=\left[\begin{array}{cc} I & 0\\ 0 & T_{1}\left(L\right) \end{array}\right]\left[\begin{array}{c} b(0)\\ a(L) \end{array}\right]=\left[\begin{array}{cc} I & 0\\ 0 & T_{1}\left(L\right) \end{array}\right]\left[\begin{array}{cc} R_{1}\left(L\right) & B(L)\\ F(L) & R_{2}\left(L\right) \end{array}\right]\left[\begin{array}{cc} I & 0\\ 0 & T_{2}^{-\;1}\left(L\right) \end{array}\right]\left[\begin{array}{c} a(0)\\ \tilde{b}\left(L\right) \end{array}\right]\\ =\left[\begin{array}{cc} R_{1}\left(L\right) & B\left(L\right)T_{2}^{-\;1}\left(L\right)\\ T_{1}\left(L\right)F\left(L\right) & T_{1}\left(L\right)R_{2}\left(L\right)T_{2}^{-\;1}\left(L\right) \end{array}\right]\left[\begin{array}{c} a(0)\\ \tilde{b}\left(L\right) \end{array}\right]\;. \end{array}$$

This clearly shows that when no light is injected at z=L—i.e., when $\tilde{b}\left(L\right)=0$—the round-trip response still reads $b\left(0\right)=R_{1}\left(L\right)a\left(0\right)$, proving the invariance of $R_{1}\left(L\right)$ to the arbitrary transformations $T_{1}$ and $T_{2}$. As discussed in Section 2.4 of the main article, this is the consequence of the rather obvious fact that $R_{1}\left(L\right)$ describes the measurement performed at z=0 and there is not any physical reason why this measurement should depend on the reference frame specified along the fiber.

The transformations $T_{1}$ and $T_{2}$ do have, however, an impact on the interpretation of the measurement. For reasons to be clarified later, we now consider the specific case in which $T_{1}\left(z\right)=T_{2}\left(z\right)=T\left(z\right)$ with T real and orthogonal, so that $T^{-\;1}=T^{T}$. Then, repeating the above calculations starting from (4) of the main article, we find

(A43) $$\left[\begin{array}{c} b(0)\\ \tilde{a}\left(z\right) \end{array}\right]=\left[\begin{array}{cc} R_{1}\left(z\right) & B\left(z\right)T^{T}\left(z\right)\\ T(z)F(z) & T\left(z\right)R_{2}\left(z\right)T^{T}\left(z\right) \end{array}\right]\left[\begin{array}{c} a(0)\\ \tilde{b}\left(z\right) \end{array}\right]\;.$$

This proves that in the transformed forward propagation matrix reads $\tilde{F}\left(z\right)=T\left(z\right)F\left(z\right)$ and varies as a function of *z* according to

(A44) $$\partial_{z}\tilde{F}=\left\{\left(\partial_{z}T\right)T^{T}-jT\left(Q+R_{2}S_{1}\right)T^{T}\right\}\tilde{F}\;.$$

The matrix Q(z) is equal to the sum of several terms due to the different perturbations acting on the fiber; one of these terms is $jK_{\mathrm{twist}}\left(z\right)$ and it is given by the twist (see Appendix A.1). Note that the *z*-dependence of $K_{\mathrm{twist}}$ is due only to the twist rate τ(z); therefore, we can write $K_{\mathrm{twist}}\left(z\right)=\tau \left(z\right)K_{0}$, where $K_{0}$ is a constant matrix whose actual structure depends on the considered modes but is of no importance for the present analysis [33,34]. We now set

(A45) $$\partial_{z}T\left(z\right)=-K_{\mathrm{twist}}\left(z\right)T\left(z\right)=-\tau \left(z\right)K_{0}T\left(z\right)\;,\;T\left(0\right)=I\;,$$

which is consistent with the starting assumption that T(z) is orthogonal because $K_{\mathrm{twist}}$ is real and anti-symmetric. Moreover, note that T(z) and $K_{0}$ commute, since the latter is constant. Owing to this definition of T, (A44) becomes

(A46) $$\partial_{z}\tilde{F}=-jT\left(Q_{\mathrm{no}-\mathrm{twist}}+R_{2}S_{1}\right)T^{T}\;\tilde{F}\;,$$

where $Q_{\mathrm{no}-\mathrm{twist}}$ includes all the perturbations, except twist.

Similarly, we have $\tilde{B}\left(z\right)=B\left(z\right)T^{T}\left(z\right)$ and

(A47) $$\partial_{z}\tilde{B}=\tilde{B}\left\{T{\left(\partial_{z}T\right)}^{T}+jT\left(H+S_{1}R_{2}\right)T^{T}\right\}\;.$$

Owing to reciprocity of twist, the corresponding term in H is $C_{\mathrm{twist}}=-K_{\mathrm{twist}}^{T}$; therefore, using (A45), we prove that the transformed backward propagation matrix also obeys the equation

(A48) $$\partial_{z}\tilde{B}=j\tilde{B}T\left(H_{\mathrm{no}-\mathrm{twist}}+S_{1}R_{2}\right)T^{T}\;,$$

where $H_{\mathrm{no}-\mathrm{twist}}$ includes all the perturbations, except twist.

The meaning of the above analysis is that round trip measurement can be thought of as being made on a fiber with no twist; in this scenario, the effect of the twist is a transformation of the coupling matrix according to the matrix T(z) given by (A45), which depends only on the twist rate τ(z). It can also be shown that the transformation T(z) corresponds to rotating the local reference frame around the fiber axis at a rate proportional to τ(z) [35].

### Appendix C.5. Proof of the Master Equations for Distributed Polarization Sensing

In order to prove Equations (33) and (34) of the main article, we start by recalling, for completeness, that the Stokes vector $\bar{S}=\left(S_{0},\;S_{1},\;S_{2},\;S_{3}\right)$ associated to the Jones vector a is defined as $\bar{S}=a^{*}\bar{\sigma }a$, where $\bar{\sigma }=\left(\sigma_{0},\;\sigma_{1},\;\sigma_{2},\;\sigma_{3}\right)$ is the vector of the Pauli matrices [157]

(A49) $$\sigma_{0}=\left[\begin{array}{cc} 1 & 0\\ 0 & 1 \end{array}\right]\;,\;\sigma_{1}=\left[\begin{array}{cc} 1 & 0\\ 0 & -1 \end{array}\right]\;,\;\sigma_{2}=\left[\begin{array}{cc} 0 & 1\\ 1 & 0 \end{array}\right]\;,\;\sigma_{3}=\left[\begin{array}{cc} 0 & j\\ -j & 0 \end{array}\right]\;.$$

An equivalent representation is given by the coherence matrix defined as $C_{a}=aa^{*}$ [58], which actually reads

(A50) $$C_{a}=\frac{1}{2}\left[\begin{array}{cc} S_{0}+S_{1} & S_{2}+jS_{3}\\ S_{2}-jS_{3} & S_{0}-S_{1} \end{array}\right]=\frac{1}{2}\sum_{n=0}^{3}S_{n}\sigma_{n}=\frac{1}{2}\bar{S}\;\cdot \;\bar{\sigma }\;.$$

Neglecting double scattering, and referring to the rotated reference frame described in Section 2.4 of the main article, the forward propagation matrix obeys the equation (see (8), (10) and (17) of the main article)

(A51) $$\partial_{z}F=-j\left[\left(\beta -j\alpha \right)I+K_{L}+jK_{F}\right]F\;,$$

where β is the propagation constant, α the attenuation coefficient, $K_{L}$ accounts for linear birefringence and twist and $K_{F}$ for Faraday rotation—these two matrices are both real. Assuming that the fiber has no PDL, we can write $F\left(z\right)=e^{-j\beta z}e^{-\alpha z}U\left(z\right)$, where U(z) is unitary with unit determinant and obeys the equation

(A52) $$\partial_{z}U=-j\left(K_{L}+jK_{F}\right)U=-jKU\;.$$

The fact that U is unitary implies that the matrix K is Hermitian, hence $K_{L}$ is symmetric and $K_{F}$ is anti-symmetric. Similarly, also the matrix of backward propagation can be factorized as $B\left(z\right)=e^{j\beta z}e^{\alpha z}V\left(z\right)$, where V(z) is unitary with unit determinant and obeys the equation

(A53) $$\partial_{z}V=-jV\left(K_{L}+jK_{F}\right)=-jVK\;.$$

Let ${\hat{a}}_{0}$ be the normalized Jones vector at the input of the fiber; the Jones vector of the forward propagating field is $a\left(z\right)=U\left(z\right){\hat{a}}_{0}$. The corresponding coherence matrix $C_{a}\left(z\right)=a\left(z\right)a^{*}\left(z\right)$ then obeys the equation

(A54) $$\partial_{z}C_{a}=-j\left(KC_{a}-C_{a}K\right)\;.$$

As described in Section 3.2 of the main article, the coherence matrix of the round-trip field b(z) reads $C_{b}\left(z\right)=V\left(z\right)C_{a}\left(z\right)V^{*}\left(z\right)$. Taking the *z*-derivative of this matrix, and using the fact that $VV^{*}=V^{*}V=I$, it can be shown that

(A55) $$\partial_{z}C_{b}=-j2\left[K_{B}\left(z\right)C_{b}\left(z\right)-C_{b}\left(z\right)K_{B}\left(z\right)\right]\;,$$

where $K_{B}\left(z\right)=V\left(z\right)K\left(z\right)V^{*}\left(z\right)$. Moreover, exploiting again $V^{*}V=I$ it is straightforward to verify that (A53) yields $\partial_{z}V=-jK_{B}V$.
